# Pharmacokinetic and Pharmacodynamic Interactions Between Anti‐Asthma Drugs and Medicinal Plants: A Comprehensive Review

**DOI:** 10.1002/ptr.70436

**Published:** 2026-09-06

**Authors:** Emine Kocyigit, Aylin Bülbül, Betul Kocaadam‐Bozkurt, Osman Bozkurt, Duygu Ağagündüz, Raffaele Capasso

**Affiliations:** ^1^ Department of Nutrition and Dietetics Ordu University Ordu Türkiye; ^2^ Department of Nutrition and Dietetics Erzurum Technical University Erzurum Türkiye; ^3^ Department of Nutrition and Dietetics Kulu Faculty of Health Sciences Konya Türkiye; ^4^ Department of Nutrition and Dietetics Gazi University Ankara Türkiye; ^5^ Department of Agricultural Sciences University of Naples Federico II Portici Italy

**Keywords:** asthma, complementary medicine, herb–drug interactions, medicinal plants, pharmacodynamics, pharmacokinetics, toxicity

## Abstract

Asthma affects 260–300 million people worldwide. About 20%–35% of patients use medicinal plants with their asthma medication, often without telling their clinicians. Disclosure rates are low; up to 42% of asthma outpatients use herbal products, with no records in their charts. Previous reviews discussed herb–drug interactions and asthma phytotherapy separately. There is no combined pharmacokinetic, pharmacodynamic, and toxicological evidence for all modern anti‐asthma agents. This review closes that gap. It draws from mechanistic, preclinical, and clinical data. It looks at eight common botanicals: *Glycyrrhiza glabra* , *Curcuma longa* , *Nigella sativa* , *Boswellia serrata* , *Tylophora indica*, *Adhatoda vasica* , *Astragalus membranaceus*, and *Piper nigrum. The review explains how these botanicals affect* cytochrome P450 isoenzymes, Phase II conjugative enzymes, and membrane transporters, including P‐glycoprotein. It also considers shared adrenergic, glucocorticoid‐receptor, and inflammatory targets of inhaled corticosteroids, β_2_‐agonists, leukotriene receptor antagonists, theophylline, and biologic agents. These interactions create clinical hazards. For example, *Hypericum perforatum* increases CYP3A4 activity by up to 11‐fold and can destabilize theophylline and corticosteroid levels. *Ephedra sinica* causes dose‐dependent sympathomimetic toxicity, which worsens β_2_‐agonist cardiovascular risk. *Glycyrrhiza glabra* may trigger pseudoaldosteronism and hypokalaemia by inhibiting 11β‐hydroxysteroid dehydrogenase type 2. *Ginkgo biloba* has been tied to bleeding events. Turmeric, previously seen as safe, now appears in drug‐induced liver injury registries. Compositional variability adds to the risk; for example, hyperforin content can range from 0.01% to 1.89%, and ephedrine content varies by more than 12‐fold across products. This review places its findings in the context of the WHO Global Traditional Medicine Strategy 2025–2034. It identifies important herb–anti‐asthma drug pairs, defines monitoring endpoints, and proposes a pharmacogenetically informed framework for safely integrating medicinal plants into asthma care.

## 1 Introduction

Asthma is a chronic inflammatory disease of the airways, defined by the Global Initiative for Asthma (GINA) as a condition characterized by variable respiratory symptoms including wheezing, shortness of breath, chest tightness, and cough together with variable expiratory airflow limitation (Papi et al. 2018; Reddel et al. 2022). Asthma remains one of the leading chronic non‐communicable diseases worldwide, with an estimated 260 million prevalent cases reported in 2021 by the Global Burden of Disease Study, underscoring its substantial and persistent global health burden, with broader estimates suggesting that nearly 300 million individuals live with the condition worldwide (Oh et al. 2025; Porsbjerg et al. 2023). The burden of asthma is unequally distributed across countries, with low‐ and middle‐income settings bearing a disproportionate share of asthma‐related morbidity and mortality; this disparity is shaped by rapid urbanization, changing environmental exposures, and persistent barriers to access to essential inhaled medicines, particularly inhaled corticosteroid‐based therapy (Mortimer et al. 2022; Rodriguez et al. 2019). In 2021, asthma was responsible for an estimated 436,000 deaths and 21.4 million disability‐adjusted life‐years worldwide; although the global age‐standardized mortality rate has declined substantially since 1990, the absolute number of people living with asthma has risen again in recent years (Porsbjerg et al. 2023; Zhang et al. 2025). Collectively, these findings emphasize the continuing public health importance of asthma, a largely manageable disease that still imposes considerable clinical and economic burden worldwide despite the availability of effective therapies (Oh et al. 2025; Porsbjerg et al. 2023; Zhang et al. 2025).

The pathophysiology of asthma involves complex interactions between genetic predisposition and environmental factors that drive chronic airway inflammation and structural remodeling (Holgate et al. 2015). In the most well‐characterized inflammatory endotype, T2‐high asthma, epithelial alarmins promote Th2‐ and ILC2‐driven responses, with these cells producing the canonical type 2 cytokines IL‐4, IL‐5, and IL‐13 (Ji and Li 2023; Pesic et al. 2025). These cytokines support IgE class switching, eosinophilic airway inflammation, goblet cell metaplasia, mucus hypersecretion, and bronchial hyperresponsiveness, while also contributing to airway remodeling (Sahnoon et al. 2025; Xie et al. 2024). With persistent epithelial injury and aberrant repair, asthma remodels the airway wall through epithelial dysfunction, subepithelial fibrosis, increased airway smooth muscle mass, and enhanced angiogenesis (Hough et al. 2020). Recognition of asthma heterogeneity, encompassing both T2‐high and T2‐low endotypes, has profoundly shaped contemporary approaches to phenotype‐directed pharmacotherapy (Hammad and Lambrecht 2021; Porsbjerg et al. 2023).

The pharmacological management of asthma follows the GINA stepwise approach, which classifies medications into controllers (maintenance therapy) and relievers across five escalating treatment steps (GINA 2024). Short‐acting β_2_‐agonists (SABAs) such as salbutamol and terbutaline have historically served as the primary relievers, providing rapid bronchodilation through β_2_‐adrenoceptor–mediated smooth muscle relaxation; however, GINA now recommends against SABA‐only treatment, as mounting evidence indicates that regular use without concomitant anti‐inflammatory therapy increases the risk of severe exacerbations and asthma‐related mortality (GINA 2024; Reddel et al. 2022). But Long‐acting β_2_‐agonists (LABAs), including salmeterol and formoterol, provide more sustained bronchodilation, but in asthma they should be prescribed only in combination with inhaled corticosteroids (ICS) rather than as monotherapy (Busse et al. 2018; Reddel et al. 2022). ICS, such as budesonide, fluticasone propionate, and beclometasone dipropionate, remain the cornerstone of controller treatment, acting through the glucocorticoid receptor to suppress inflammatory gene transcription, including nuclear factor‐κB (NF‐κB)–dependent pathways, in part through histone deacetylase 2 mediated mechanisms (Barnes 2010; Kim and Agrawal 2025). Accordingly, ICS/LABA combinations form the mainstay of asthma treatment from Step 3 onward, offering complementary anti‐inflammatory and bronchodilator effects and benefiting from well‐described pharmacological synergy, including corticosteroid‐induced upregulation of β2‐receptor expression and LABA‐enhanced glucocorticoid receptor nuclear translocation (Barnes 2010; Reddel et al. 2022).

Additional options within asthma pharmacotherapy include leukotriene receptor antagonists (LTRAs), such as montelukast and zafirlukast, which target the cysteinyl leukotriene pathway and may be particularly useful in selected settings, including exercise‐induced bronchoconstriction and, in some patients, aspirin‐exacerbated respiratory disease (Barnes 2006). Among anticholinergic agents, ipratropium is used mainly as an adjunct during acute exacerbations, whereas tiotropium has an established role as add‐on maintenance therapy in patients who remain symptomatic despite ICS/LABA treatment; these drugs attenuate cholinergic bronchoconstriction, and tiotropium produces bronchodilation through inhibition of airway smooth‐muscle M3 receptors (GINA 2024; Gosens and Gross 2018). According to GINA, theophylline is not recommended for the management of acute asthma exacerbations because of its limited efficacy, unfavorable safety profile, and the preference for more appropriate bronchodilator therapy, particularly SABAs (GINA 2024). For severe asthma that remains uncontrolled despite optimized conventional therapy, biologic agents have substantially expanded treatment options: omalizumab targets IgE, mepolizumab targets IL‐5, and dupilumab blocks IL‐4 receptor alpha, with evidence supporting reductions in exacerbations and, particularly for dupilumab, oral corticosteroid requirements in appropriate patients (Gyawali et al. 2025). Importantly, several medications used in asthma management rely on cytochrome (CYP) P450–mediated metabolism. Theophylline is metabolized mainly by CYP1A2, whereas inhaled corticosteroids such as budesonide and fluticasone propionate are cleared predominantly via CYP3A4 (Barnes 2011; Lipworth and Dagg 1994). This enzymatic dependence renders these agents particularly susceptible to clinically significant pharmacokinetic drug interactions.

Alongside guideline‐based pharmacotherapy, many patients with asthma continue to use complementary approaches, particularly herbal medicines (HMs) (Amaral‐Machado et al. 2020). The World Health Organization (WHO) has recognized that approximately 80% of the global population utilizes traditional medicine for some aspect of primary healthcare (World Health Organization 2013). Among the herbal products repeatedly examined in the asthma literature are *Nigella sativa* , *Boswellia serrata* , *Curcuma longa* , and *Zingiber officinale* (Amaral‐Machado et al. 2020). Current evidence suggests that these agents may modulate inflammatory and airway‐response pathways including NF‐κB signaling, leukotriene‐related pathways, cytokine responses, oxidative stress, and airway smooth‐muscle reactivity but for most products the evidence still derives largely from preclinical studies or relatively small and heterogeneous clinical trials (Amaral‐Machado et al. 2020; Rajizadeh et al. 2024; Zafrilla et al. 2025). Several medicinal plants have been extensively investigated for their potential role in asthma management. Among the most studied, *Nigella sativa* contains thymoquinone as its principal bioactive constituent, which has been shown to attenuate airway inflammation through inhibition of NF‐κB signaling and suppression of 5‐lipoxygenase–mediated leukotriene biosynthesis, alongside demonstrable bronchodilatory effects (Koshak, Koshak, and Heinrich 2017; Koshak, Wei, et al. 2017). *Boswellia serrata* is another widely recognized anti‐asthmatic herb; its pentacyclic triterpenic acids, collectively known as boswellic acids, function as specific, non‐redox inhibitors of 5‐lipoxygenase, and placebo‐controlled clinical trials have confirmed their efficacy in alleviating bronchial asthma symptoms (Ammon 2006; Safayhi et al. 1992). The terpene lactones of *Ginkgo biloba* , particularly ginkgolide B, serve as potent and selective antagonists of the platelet‐activating factor receptor, a mediator implicated in airway hyperresponsiveness (Biernacka et al. 2023; Vogensen et al. 2003). *Glycyrrhiza glabra* (licorice) exerts corticosteroid‐potentiating effects primarily by inhibiting the enzyme 11β‐hydroxysteroid dehydrogenase, thereby prolonging the local activity of endogenous glucocorticoids (Izzo and Ernst 2009). *Curcuma longa* , through its major curcuminoid curcumin, exhibits broad anti‐inflammatory activity mediated by suppression of both NF‐κB–dependent transcription and cyclooxygenase‐2 expression (Lelli et al. 2017). Beyond these, several other botanicals merit attention: *Ephedra sinica* , which provided the pharmacological template for modern sympathomimetic bronchodilators through its alkaloid ephedrine; *Petasites hybridus* (butterbur), valued for its combined anti‐leukotriene and antihistaminic properties; *Zingiber officinale* (ginger), which has been shown to attenuate Th2‐mediated immune responses in experimental asthma models; and *Tylophora indica*, whose principal alkaloid tylophorine demonstrates mast cell–stabilizing and anti‐allergic activity (Huntley and Ernst 2000; Khan et al. 2015; Shivpuri et al. 1972). Collectively, these herbal agents act through diverse yet frequently overlapping pathways including anti‐inflammatory, bronchodilatory, antioxidant, and immunomodulatory mechanisms (Clark et al. 2010; De Smet 2002). The growing appeal of such remedies among patients can be attributed to perceptions of greater safety, deep‐rooted cultural and familial traditions, dissatisfaction with the side‐effect profiles of conventional pharmacotherapy, and relative affordability (George and Topaz 2013; Safayhi et al. 1992).

However, the concurrent use of HMs with conventional anti‐asthma drugs raises substantive concerns regarding pharmacokinetic and pharmacodynamic interactions that may compromise therapeutic efficacy or patient safety (Izzo and Ernst 2009; Zhou et al. 2003). Pharmacokinetic herb–drug interactions occur when phytochemicals alter the absorption, distribution, metabolism, or excretion of co‐administered pharmaceuticals, most critically through modulation of CYP450 enzymes and membrane drug transporters (Cho and Yoon 2015; Izzo and Ernst 2009). Within the CYP450 superfamily, CYP3A4, CYP1A2, CYP2C8, and CYP2C9 are of particular relevance to anti‐asthma drug biotransformation, and various herbal constituents have been shown to act as either inhibitors or inducers of these isoforms (Filppula et al. 2011; Ha et al. 1995). P‐glycoprotein (P‐gp), an ATP‐dependent efflux transporter expressed in the intestinal epithelium, hepatocytes, and renal tubular cells, is likewise modulated by a range of phytochemicals; however, this mechanism is primarily relevant to orally administered anti‐asthma agents, as inhaled formulations largely bypass intestinal P‐gp (Cho and Yoon 2015; Wessler et al. 2013). Several well‐characterized examples illustrate the clinical significance of these interactions. *Hypericum perforatum* (St. John's wort) is a potent inducer of CYP3A4 and P‐gp through activation of the pregnane X receptor (PXR) (Nicolussi et al. 2020). Although a case report documented reduced theophylline plasma concentrations during concomitant use, a subsequent controlled pharmacokinetic study in healthy volunteers found no statistically significant interaction, and the clinical relevance of this effect remains uncertain (Borrelli and Izzo 2009; Morimoto et al. 2004). Curcumin has been reported to inhibit CYP3A4 (IC_50_ = 16.3 μM) and CYP2C9 (IC_50_ = 4.3 μM) in human recombinant enzyme assays, though more recent studies using different substrates and enzyme preparations have yielded somewhat higher values, underscoring the assay‐dependent nature of such estimates (Appiah‐Opong et al. 2007; Volak et al. 2008). These findings carry potential implications for the metabolism of CYP3A4‐substrate corticosteroids and the CYP2C9‐dependent leukotriene receptor antagonist zafirlukast (Appiah‐Opong et al. 2007; Savidge et al. 1998). Thymoquinone, the principal bioactive constituent of *Nigella sativa* , has been identified as an inhibitor of CYP2C9 based on comprehensive kinetic analyses performed in human liver microsomes using warfarin and phenytoin as probe substrates (Wang et al. 2022b, 2022a). Given that zafirlukast is primarily metabolized by CYP2C9, this inhibition may be clinically meaningful; montelukast, by contrast, is predominantly a CYP2C8 substrate and is therefore less susceptible to CYP2C9‐mediated interactions (Filppula et al. 2011; Karonen et al. 2012). Glycyrrhizin merits particular attention in the context of asthma pharmacotherapy. Its active metabolite, 18β‐glycyrrhetinic acid, is a potent inhibitor of 11β‐hydroxysteroid dehydrogenase type 2, the enzyme responsible for the inactivation of cortisol in mineralocorticoid‐responsive tissues (Omar et al. 2012). This inhibition potentiates both endogenous and exogenous glucocorticoid activity and elevates the risk of hypokalemia through a pseudoaldosteronism mechanism a hazard that is compounded in asthma patients by the hypokalemic effects of β_2_‐agonists, which promote intracellular potassium shift via Na^+^/K^+^‐ATPase stimulation, and theophylline, which induces catecholamine‐mediated potassium redistribution (Hsu and Bajaj 2025; Murray 2020).

Pharmacodynamic interactions between HMs and anti‐asthma drugs may be additive, synergistic, or antagonistic (Dülger 2012). Herbal agents possessing bronchodilatory properties, such as ephedrine‐containing preparations, may produce additive effects with β_2_‐agonists, amplifying the risk of tachycardia and other cardiovascular adverse effects (Huffman 2002). Conversely, the anti‐inflammatory properties of curcumin, boswellic acids, and thymoquinone may synergize with corticosteroid therapy, potentially enabling dose reduction but also complicating the clinical assessment of therapeutic responses and increasing the risk of over immunosuppression (Abidi et al. 2014; Ahmed and Cevher 2019; Ferrara et al. 2015). The interaction potential is most critical for theophylline, whose narrow therapeutic window means that even modest perturbations in hepatic clearance, whether through CYP1A2 induction by St. John's wort or inhibition by piperine, can precipitate either therapeutic failure or life‐threatening toxicity (Morimoto et al. 2004).

Despite the evident clinical importance of herb–drug interactions in asthma pharmacotherapy, the current literature exhibits several critical deficiencies that this review seeks to address. Currently, no systematic review has specifically and comprehensively examined the pharmacokinetic and pharmacodynamic interactions between commonly used medicinal plants and anti‐asthma drugs. The available evidence is overwhelmingly preclinical, derived principally from in vitro enzyme inhibition assays and animal pharmacokinetic studies whose translational relevance to clinical practice remains uncertain (Braunwalder et al. 2025). Standardized dosing protocols, quality‐control measures, and formulation standards for herbal products used in asthma are largely absent, introducing substantial variability that complicates the prediction of interaction magnitude and clinical significance (Lin et al. 2019). Adverse events arising from herb–drug interactions are significantly underreported, partly because patients frequently do not disclose herbal medicine use to their healthcare providers and partly because existing pharmacovigilance systems were designed primarily for synthetic pharmaceuticals and require modification to adequately capture herb–drug interactions (HDIs) (Mengie et al. 2025; Shaw et al. 2012). The specific CYP450 and transporter‐mediated interaction profiles for many commonly used anti‐asthma herbs remain insufficiently characterized, with robust human pharmacokinetic data available for only a small number of phytochemical–drug combinations (Fasinu et al. 2012). Geographic disparities further limit the evidence base, as the majority of studies originate from Asian and African traditional medicine contexts, with limited randomized clinical trial data from Western populations (Kacholi 2024; Shergis et al. 2016). Evidence‐based guidelines for healthcare providers on the safe management of patients who concurrently use HMs and conventional anti‐asthma pharmacotherapy remain conspicuously absent.

This comprehensive review aims to bridge these gaps by systematically evaluating the available preclinical, clinical, and mechanistic evidence on pharmacokinetic and pharmacodynamic interactions between anti‐asthma drugs and medicinal plants. By critically appraising the molecular basis and clinical significance of these interactions, identifying specific high‐risk drug–herb combinations, and delineating priority areas for future investigation, this review seeks to contribute to the development of evidence‐based recommendations for the safe and rational integration of HMs within contemporary asthma management frameworks.

## 2 Overview of Anti‐Asthma Drugs

The development of asthma treatment first concentrated on bronchodilators, especially adrenergic agonists, which were implemented to alleviate bronchoconstriction and have been utilized therapeutically for about a century (Kapri et al. 2023). The acknowledgment of chronic airway inflammation's pivotal role in asthma etiology resulted in the implementation of inhaled corticosteroids (ICS) in the 1970s as the foundation of long‐term therapy, markedly decreasing severe exacerbations. The advancement of metered‐dose inhalers (MDIs) enhanced treatment by facilitating precise drug distribution and minimizing systemic side effects (Tanaka 2015).

In the early 20th century, available asthma treatments included oral ephedrine, intravenous adrenaline, inhaled epinephrine (sympathomimetic agents), inhaled scopolamine (anticholinergics), and oral caffeine or theophylline (methylxanthines), all designed to alleviate acute and life‐threatening airway obstruction (Brusselle and Koppelman 2022). Nonetheless, they failed to address the etiology of asthma and did not offer sustained therapeutic benefits (Lommatzsch et al. 2022). Current asthma pharmacotherapy incorporates classic bronchodilators and ICS, as well as long‐acting beta‐agonists (LABA), leukotriene receptor antagonists (LTRA), and, more recently, biological agents. Monoclonal antibodies (e.g., omalizumab, mepolizumab, benralizumab) particularly target interleukin‐5 or IgE in severe, uncontrolled asthma, therefore regulating the inflammatory response and enhancing clinical results (Zhen et al. 2022).

Recent studies and advancements in technology have led to a greater personalization of inhalation therapies (Varricchi et al. 2025; Xie et al. 2024). Currently, safe and effective treatments are provided that target the inflammatory processes underlying asthma, aiming to prevent symptoms and exacerbations, and incorporating phenotype‐specific immunomodulators. These medications effectively regulate inflammation and enhance the functionality of structural cells, including epithelial cells, smooth muscle cells, fibroblasts, and nerve cells (Picado et al. 2025).

Asthma guidelines updated treatment recommendations in accordance with novel therapies. The GINA guidelines have eliminated short‐acting beta‐2 agonist (SABA) monotherapy, methylxanthines, and systemic glucocorticoids from the treatment steps, substituting them with phenotype‐specific, anti‐inflammatory therapies aimed at preventing acute symptoms and exacerbations, as well as reducing mortality rates and future risks (Reddel et al. 2022). The GINA recommends against making only use of SABA for treating asthma in adults, adolescents, or children aged 6–11 years. It is believed that treatments incorporating ICS should be utilized to mitigate the risk of severe exacerbations and manage symptoms (Venkatesan 2025). GINA developed two primary therapy methods for adults and adolescents in its asthma management strategy. Track 1, which employs low‐dose ICS‐formoterol as a reliever, is endorsed as the optimal strategy; in this framework, patients utilized low‐dose ICS‐formoterol upon the onset of symptoms and, in more advanced stages, administered the same medication for both maintenance and relief (MART). This method has been documented to markedly diminish the likelihood of severe exacerbations in comparison to SABA‐based therapies, deliver comparable or superior symptom management, and present the convenience of a single inhaler for therapy. Track 2 is designated as an alternative strategy utilizing SABA or ICS‐SABA as a reliever, favored when Track 1 is inapplicable or when the patient is stable. However, it is underscored that inadequate adherence to ICS treatment may lead patients to rely solely on SABA, thereby elevating the risk of exacerbation. The principal aim throughout all phases of treatment is to avert major flare‐ups; this strategy alleviates the strain on both the patient and the healthcare system, while concurrently minimizing the necessity for oral corticosteroids, which have long‐term adverse consequences (Global Initiative for Asthma 2025).

Maintenance medication for asthma serves as the primary pharmaceutical strategy to mitigate chronic airway inflammation and diminish the likelihood of long‐term exacerbations. ICS constitute the foundation of this therapy. ICSs modulate nuclear transcription by interacting with glucocorticoid receptors, suppress proinflammatory transcription factors including NF‐κB and AP‐1, and diminish the expression of cytokines like IL‐4, IL‐5, and IL‐13. This process led to the suppression of eosinophilic inflammation and a reduction in airway hyperreactivity (Papi et al. 2018). From a pharmacokinetic perspective, it was documented that ICS are predominantly metabolized in the liver through CYP3A4, and that systemic corticosteroid exposure is heightened when administered alongside powerful CYP3A4 inhibitors (Sun et al. 2025).

Controlling therapies were consistently employed to manage asthma symptoms over the long term and prevent potential risks. This group predominantly comprised inhaled corticosteroids and ICS‐LABA combinations. LABA were used in conjunction with ICS because, despite their ability to relax bronchial smooth muscle by activating β‐adrenergic receptors, they had no direct effect on the inflammatory process (Martinez‐Tenopala et al. 2025). The ICS components in combination therapy are generally metabolized by CYP3A4, while formoterol metabolism is mainly via conjugation, with minimal CYP involvement (Shang et al. 2022). LTRA are a distinct category of controller medicines, especially advantageous for allergic and eosinophilic asthma phenotypes. Montelukast, the predominant LTRA, functions by inhibiting the binding of cysteinyl leukotrienes to the CysLT_1_ receptor, thereby diminishing asthma and inflammation of the airways. Montelukast is metabolized hepatically, predominantly via CYP2C8, with supplementary involvement from CYP2C9 and CYP3A4 (Mizernitskiy and Petrova 2024).

Reliever drugs were employed to swiftly mitigate acute bronchoconstriction. Within this group, SABA were predominantly favored. SABA induced bronchodilation within minutes through β_2_‐receptor activation, however they did not directly influence the inflammatory process (Papi et al. 2018). The primary metabolic pathway of SABA medicines, including salbutamol, is Phase II sulfate conjugation, predominantly facilitated by the SULT1A3 enzyme, while the role of CYP450 enzymes is secondary (Marques and Vale 2022).

The anti‐inflammatory reliever (AIR) technique uses a mix of low‐dose inhaled corticosteroids and formoterol, which works swiftly. This method involved inhaled corticosteroids inhibiting inflammatory gene expression, while formoterol facilitated fast bronchodilation by β_2_‐adrenergic receptor stimulation. Clinical investigations showed that reliever therapy utilizing ICS‐formoterol when needed considerably diminishes the incidence of severe asthma exacerbations compared to traditional SABA. Post hoc analyses found that as‐needed ICS‐formoterol treatment diminished the incidence of severe exacerbations while preserving asthma control levels (Hatter et al. 2022). Comprehensive analyses with moderate to high levels of evidence corroborated this finding, demonstrating that the ICS/formoterol approach was more efficacious than SABA in mitigating the risk of severe exacerbation (Beasley et al. 2024; Krishnan and Buhl 2024). A meta‐analysis indicated that ICS‐formoterol diminished the occurrence of severe exacerbations in comparison to SABA, corroborating the findings of all trials (Rayner et al. 2025).

MART has been established as an integrative therapeutic strategy utilizing the same ICS‐formoterol inhaler for both maintenance and symptomatic relief. Randomized controlled trials showed that the MART method reduced the incidence of severe flare‐ups and improved treatment adherence relative to fixed‐dose regimens (Beasley et al. 2019; O'Byrne et al. 2018). Budesonide, utilized in the MART treatment, is metabolized by CYP3A4, while formoterol undergoes primarily CYP‐independent conjugation, resulting in a reduced risk of drug–drug interactions (DDIs). Furthermore, the principal metabolic pathway for formoterol, a long‐acting beta_2_‐agonist, is glucuronidation, with CYP450 isoenzymes (CYP2D6, CYP2C19, CYP2C9, CYP2A6) contributing minimally to this process (Carr et al. 2023; discovery).

Table 1 summarizes the pharmacological agents, mechanisms of action, and metabolic pathways involved in asthma treatment strategies, thereby providing a mechanistic framework for understanding potential HDIs. Anti‐asthma drugs metabolized by CYP3A4, sulfotransferases (SULTs), or conjugation pathways may exhibit increased vulnerability to variations in systemic drug exposure and therapeutic efficacy when administered with herbal remedies that inhibit or promote these enzymes. Monoclonal antibodies that are not eliminated through cytochrome P450 enzymes or membrane transporter proteins are typically regarded as having restricted potential for direct participation in pharmacokinetic DDIs. A thorough evaluation of the pharmacological profiles of anti‐asthma drugs is essential for anticipating and effectively controlling possible interactions that may occur when administered simultaneously with HMs.

**TABLE 1:** Pharmacological agents, mechanisms of action, and metabolic pathways involved in asthma treatment strategies.

Pharmacological agents	Mechanism of action	Enzyme systems
Maintenance treatment (Carr et al. 2023; Rayner et al. 2025) ICS	Bind to cytoplasmic glucocorticoid receptors, regulate gene transcription NF‐κB and AP‐1 activation 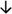 Production of IL‐4, IL‐5, and IL‐13 levels 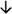 Expression of anti‐inflammatory proteins 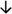 Eosinophilic inflammation, airway oedema and hyperreactivity	CYP3A4
Controller (Noorduyn et al. 2025; Society 2025) ICS‐LABA combinations LTRA Biological agents (e.g., anti‐IL‐5 or anti‐IgE antibodies)	ICS bind to cytoplasmic glucocorticoid receptors, suppresses the expression of proinflammatory cytokines and chemokines İnhibition of NF‐κB and AP‐1 levels LABA stimulate β_2_‐adrenergic receptors, activating the adenylate cyclase enzyme, cAMP levels LTRA specifically montelukast and zafirlukast, mitigate the effects of leukotrienes Biological agents, such as anti‐IL‐5 or anti‐IgE monoclonal antibodies, selectively interact with cytokines through unique mechanisms that target proinflammatory mediators and regulate the inflammatory response	CYP3A4 CYP2D6 CYP2C19 CYP2C9 CYP2A6 Proteolytic degradation independent of the CYP450 system CysLT_1_ receptor antagonism
Reliever (Marques and Vale 2022, 2024) SABA	SABA binds to β_2_‐adrenergic receptors, activating the adenylate cyclase enzyme, 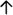 cAMP levels	SULT‐mediated sulfate conjugation CYP450
AIR (Beasley et al. 2024; Levy and Crooks 2024) Combination of low‐dose ICS and fast‐acting beta‐2 agonist formoterol (budesonide + formoterol preparations)	ICS bind to cytoplasmic glucocorticoid receptors, suppresses the expression of proinflammatory cytokines and chemokines Formoterol activates β_2_‐adrenergic receptors, promotes relaxation of the smooth muscles	CYP3A4, CYP2D6, CYP2C19, CYP2C9, CYP2A6 Glucuronidation and conjugation
MART (Cusack et al. 2020; Kim et al. 2022) (Budesonide‐formoterol combination therapy)	Budesonide functions as an ICS to mitigate chronic airway inflammation, while formoterol delivers fast bronchodilation as a β_2_‐adrenergic receptor agonist. Budesonide bind to cytoplasmic glucocorticoid receptors, suppresses the expression of proinflammatory cytokines 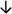 Activity of inflammatory cells	CYP3A4 Glucuronidation O‐demethylation

Abbreviations: AIR, anti‐inflammatory reliever; AP‐1, activator protein 1; cAMP, cyclic adeno‐sine monophosphate; CYP, cytochrome; ICS, inhaled corticosteroids; IL, interleukin; LABA, long‐acting beta‐agonists; LTRA, leukotriene receptor antagonists; MART, maintenance and reliever therapy; NF‐κB, nuclear factor kappa B; SABA, short‐acting beta‐2 agonist.

## 3 Herbal Medicines Used in Asthma Treatment

Asthma is a respiratory disease that develops as a result of chronic inflammation. Corticosteroid medications are widely used in the treatment of this condition (Aulidaweli 2023). However, in addition to these drugs, it has been reported that certain medicinal plants, which have been used for many years in traditional medicine particularly in countries such as India, China, Iran, and Korea, are employed in the treatment of respiratory diseases (Campbell 2025). It has been demonstrated that plants such as *Glycyrrhiza glabra* , *Tylophora indica*, *Curcuma longa* , *Adhatoda vasica* , and *Nigella sativa* may be beneficial in the treatment of asthma through various active constituents present in their composition. It is thought that these plants may contribute to disease management by suppressing the elevated inflammation observed in asthma due to their anti‐inflammatory properties (Usmani et al. 2023). However, although numerous plant constituents are used in traditional medicine for the treatment of asthma, there is an insufficient number of studies evaluating their potential side effects. Further studies are needed to adequately assess the effects of these plants. Medicinal plants used in asthma treatment are presented in Table 2.

**TABLE 2:** Medicinal plants used in asthma treatment.

Plant name	Bioactive compound	Mechanism of action	Clinical evidence level
*Glycyrrhiza glabra* (Guftar et al. 2026; Hasan et al. 2021; Sadek et al. 2020)	Glycyrrhizin, glabridin, 18β‐glycyrrhetinic acid	Anti‐inflammatory, immunomodulatory, bronchodilatory, antioxidant; suppresses Th2 cytokines, reduces IgE and eosinophils	Moderate–high (randomized clinical and animal studies)
*Tylophora indica* (Gururani et al. 2023; Sheeba et al. 2025)	Tylophorine, tylophorinidine	Anti‐inflammatory, immunomodulatory, mast cell stabilization, bronchodilatory	Moderate (clinical and preclinical studies)
*Curcuma longa* (Abidi et al. 2014; El‐Saadony et al. 2025; Kim et al. 2011; Manarin et al. 2019)	Curcumin	Anti‐inflammatory, antioxidant, inhibits histamine release, reduces airway remodeling	Moderate–high (randomized controlled clinical trial)
*Adhatoda vasica (Vasaka*) (Gujjarwar 2025; Jain et al. 2024)	Vasicine, vasicinone, adhatodine	Bronchodilatory, anti‐inflammatory, antioxidant, mast cell stabilization	Moderate (preclinical evidence)
*Nigella sativa* (Barlianto et al. 2018; Ghobadi et al. 2025; Mohammadzadeh et al. 2025)	Thymoquinone, nigellone	Anti‐inflammatory, bronchodilatory, antioxidant, immunomodulatory	Moderate (clinical and animal studies)
*Boswellia serrata* (Yugandhar et al. 2018)	Boswellic acids	Anti‐inflammatory, leukotriene synthesis inhibitor	Moderate (randomized controlled clinical trial)
*Inula japonica* (Jung et al. 2023; Li, Guo, et al. 2023)	1,10‐Seco‐eudesmanolide derivatives, flavonoids	Anti‐inflammatory, suppresses PI3K/AKT/MEK/ERK and JAK–STAT pathways	Low–moderate (preclinical and animal studies)
*Astragalus membranaceus* (Li, Liu, et al. 2022; Wei et al. 2019)	Polysaccharides, astragaloside	Anti‐inflammatory, immunomodulatory, improvement of lung function	Low–Moderate (preclinical and rat studies)
*Justicia adhatoda* ( *Adhatoda vasica* ) (Gheware et al. 2021)	Vasicine, vasicinone, flavonoids	Bronchodilatory, anti‐inflammatory, antioxidant, mast cell stabilization	Moderate (traditional, preclinical evidence, rat studies)
*Piper nigrum* L. (Alateeqi et al. 2024; Bui et al. 2017; Pei et al. 2020)	Novel amide alkaloids	Inhibit NF‐κB activation via IKK‐β, ↓ iNOS, NO, IL‐1β, IL‐6, TNFα, PGE_2_ in macrophages; anti‐inflammatory in paw edema	Moderate (traditional and rat studies)
*Glycyrrhiza glabra* (Sherwani and Nawab 2024) (Guftar et al. 2026; Hasan et al. 2021; Sadek et al. 2020)	Glycyrrhizin, glabridin, 18β‐glycyrrhetinic acid	Anti‐inflammatory, immunomodulatory, bronchodilatory, antioxidant; suppresses Th2 cytokines, reduces IgE and eosinophils	Moderate–High (randomized clinical and animal studies)
*Tylophora indica* (Gururani et al. 2023; Sheeba et al. 2025)	Tylophorine, tylophorinidine	Anti‐inflammatory, immunomodulatory, mast cell stabilization, bronchodilatory	Moderate (clinical and preclinical studies)
*Curcuma longa* (Abidi et al. 2014; El‐Saadony et al. 2025; Kim et al. 2011; Manarin et al. 2019)	Curcumin	Anti‐inflammatory, antioxidant, inhibits histamine release, reduces airway remodeling	Moderate–high (randomized controlled clinical trial)

Abbreviations: AKT, protein kinase B; ERK, extracellular signal‐regulated kinase; IgE, immunoglobulin E; IKK‐β, IκB kinase beta; IL‐1β, interleukin 1 beta; IL‐6, interleukin 6; iNOS, inducible nitric oxide synthase; JAK, Janus kinase; MEK, mitogen‐activated protein kinase kinase; NF‐κB, nuclear factor kappa‐light‐chain‐enhancer of activated B cells; NO, nitric oxide; PGE_2_, prostaglandin E2; PI3K, phosphoinositide 3‐kinase; STAT, signal transducer and activator of transcription; Th2, T helper 2 cells; TNF‐α, tumor necrosis factor alpha.

### 3.1 *Glycyrrhiza glabra*

Licorice root, known as *Glycyrrhiza glabra* , belongs to the Leguminosae family. *Glycyrrhiza glabra* is frequently used for therapeutic purposes in Ayurveda. This medicinal plant is found not only in Asia but also in certain regions of Europe (Wahab et al. 2021). This plant, which is predominantly cultivated in Asia, is also found in certain regions of Europe (Sherwani and Nawab 2024).

*Glycyrrhiza glabra* exhibits antihistaminic activity through its glycyrrhizin and flavonoid content, thereby suppressing allergic reactions and reducing mucus secretion, which plays a significant role in the management of asthma. Additionally, due to its anti‐inflammatory and bronchodilatory effects, it reduces airway inflammation and facilitates breathing (Shinu et al. 2023). Licochalcone A, obtained from the root extract of the plant, suppresses the activation of the IκB kinase complex and NF‐κB induced by TNF‐α. Various flavonoids present in the plant exhibit anti‐inflammatory effects by reducing Ig levels, IL‐3, IL‐5, and IL‐13 concentrations, decreasing eosinophilic lung inflammation, and increasing IFN‐γ activity (Hasan et al. 2021).

In a study conducted by Guftar et al. using an OVA‐induced allergic asthma mouse model, the anti‐inflammatory and immunomodulatory effects of liquiritin extract obtained from *Glycyrrhiza glabra* L. were evaluated. It was demonstrated that liquiritin administration significantly reduced lung edema, airway inflammation, and eosinophilic infiltration compared to the control group. In addition, it was reported that the mRNA expression of the Th2‐associated cytokines IL‐4 and IL‐5, which play a key role in the pathogenesis of allergic asthma, was significantly suppressed following liquiritin treatment (Guftar et al. 2026).

In a clinical study conducted by Sadek et al. in patients with asthma, the efficacy and safety of licorice (liquorice) extract were evaluated. A total of 127 asthma patients were enrolled in the study and were divided into three groups receiving placebo, 500 mg of liquorice twice daily, or 500 mg of liquorice three times daily, in addition to inhaled corticosteroid and long‐acting β_2_‐agonist therapy. At the end of the 4‐week intervention, liquorice treatment resulted in significant improvements in forced vital capacity %, forced expiratory volume in 1 s (FEV_1_) %, and asthma control test scores. These findings indicate that liquorice may provide potential benefits as an adjunct therapy in asthma (Sadek et al. 2020).

### 3.2 Curcuma longa

*Curcuma longa* L. (turmeric, Zingiberaceae) is a plant that has been used in traditional medicine for thousands of years and is widely distributed in tropical regions such as Asia and Brazil. It is commonly cultivated in tropical regions including Asia and Brazil. The roots of *C. longa* have long been used in traditional medicine for the treatment of respiratory diseases. The most well‐known health‐related effect of curcumin is its ability to regulate inflammatory responses and cytokine release by suppressing NF‐κB activation through inhibition of NF‐κB inhibitor alpha (I‐κBα) phosphorylation and degradation, as well as blocking the phosphorylation of I‐κBα kinase (IKKα) (El‐Saadony et al. 2025). Due to these anti‐inflammatory effects, it has been suggested that it may be beneficial in alleviating asthma symptoms. In a randomized controlled study conducted by Manarin et al., the effects of administering approximately 30 mg/kg/day of *Curcuma longa* root powder for 6 months, in addition to standard therapy, were evaluated in children and adolescents aged 7–18 years with asthma. At the end of the study, it was shown that the group receiving *Curcuma longa* had lower use of short‐acting β_2_‐agonists and better asthma control compared to the placebo group; however, no significant difference was observed between the groups in terms of FEV_1_ values. No serious treatment‐related adverse effects were reported (Manarin et al. 2019).

In addition to these findings, another randomized controlled study administered curcumin (1000 mg twice daily; *n* = 9) or placebo (*n* = 6) for 6 months to individuals with allergic asthma. Following curcumin administration, no significant differences were observed in asthma‐related parameters such as FEV_1_, serum IgE levels, or blood leukocyte and eosinophil counts (Kim et al. 2011). *Curcuma longa* has significant effects in alleviating inflammation. However, there are varying research outcomes regarding its effectiveness in the treatment of asthma.

### 3.3 Tylophora Indica

The leaves of *Tylophora indica* (Burm. f.) Merr. ( *T. indica* ) are frequently used in Ayurvedic traditional medicine for the treatment of a wide range of diseases, including respiratory disorders, epilepsy, diarrhea, and microbial infections. It is used for the treatment of diseases in many different regions such as Australia, Africa, and the Malay Archipelago (Gururani et al. 2020; Kumar et al. 2019). The chloroform fraction (F‐2) obtained from the metabolic extract of *Tylophora indica* leaves has been identified as the most effective fraction. This fraction has been reported to exhibit strong affinity for histamine receptors and therefore may be beneficial in the treatment of allergic asthma due to its antihistaminic effects (Gururani et al. 2023; Urolagin et al. 2024).

In a rat study investigating the relationship between asthma and the F‐2 fraction obtained from *Tylophora indica* leaves, it was determined that the F‐2R fraction inhibited contractions induced by histamine, acetylcholine, and KCl by exerting noncompetitive antagonism at histamine and muscarinic receptors and activating the β_2_‐adrenergic receptor and the sGC/cGMP signaling pathway. In an in vivo OVA‐induced asthma model, administration of the F‐2 fraction improved impairments in specific airway resistance (sRaw) and dynamic lung compliance (Cdyn).

Additionally, reductions in leukocyte and eosinophil counts as well as decreases in IgE, IL‐4, IL‐13, and TNF‐α levels were observed. Histopathological examinations demonstrated reduced airway inflammation and bronchial wall thickening. Acute toxicity studies reported that F‐2 was safe at doses of up to 2000 mg/kg (Gururani et al. 2023). These findings support the traditional antiasthmatic effects of *Tylophora indica*.

### 3.4 *Adhatoda vasica* (Vasaka)

*Adhatoda vasica* Nees belongs to the Acanthaceae family and is also known as vasaka, adosa, and Malabar nut tree. The plant is recognized as one of the medicinal herbs used in Ayurvedic and Unani traditional medicine (Annamalai Thanigaivel et al. 2017). *Adhatoda vasica* , an evergreen plant, is widely distributed in certain regions of India, Sri Lanka, and Myanmar. In the Ayurvedic medical system, *Adhatoda vasica* is regarded as one of the primary medicinal plants for the treatment of respiratory diseases such as cough, common cold symptoms, asthma, and bronchitis (Annamalai Thanigaivel et al. 2017; Choudhary et al. 2021).

*Adhatoda vasica* exhibits bronchodilatory effects by relaxing bronchial smooth muscles and increasing airflow. Through its expectorant properties, it facilitates the clearance of mucus from the respiratory tract. Additionally, *Adhatoda vasica* possesses anti‐inflammatory properties and exhibits antimicrobial activity against pathogens present in the respiratory tract (Gujjarwar 2025). In a clinical study conducted by Jain et al., the clinical efficacy of the Vasa Kantakari Kwatha formulation, containing equal proportions of *Adhatoda vasica* and *Solanum virginianum* (Kantakari), was evaluated in children with asthma. The study included 10 children aged 5–12 years, and the treatment was administered orally for 30 days. Subjective symptoms such as cough, wheezing, dyspnea, use of accessory respiratory muscles, and sleep disturbance, as well as objective parameters including complete blood count, erythrocyte sedimentation rate, and FEV_1_, were analyzed before and after treatment. At the end of the study, marked improvements were observed in symptoms such as cough (55.55%), wheezing (50%), dyspnea (53.57%), and sleep disturbance (68%), along with statistically significant improvements in eosinophil count, ESR, and FEV_1_ values (Jain et al. 2024). Although there are a limited number of studies evaluating the effects of *Adhatoda vasica* on asthma, the outcomes of the available studies are promising.

### 3.5 *Nigella sativa*

*Nigella sativa* , belonging to the Ranunculaceae family, is widely distributed across many regions including the Mediterranean, Southwest Asia, Southern Europe, North Africa, India, Pakistan, and various parts of Iran. Also known as black cumin, *Nigella sativa* is commonly used for conditions such as nasal congestion, cough, headache, asthma, and allergic disorders (Ahmad et al. 2021). *Nigella sativa* contains numerous chemical constituents such as saponins, alkaloids, fixed and volatile oils, as well as various fatty acids including linoleic, oleic, palmitic, dihomolinolenic, eicosadienoic, myristic, arachidonic, and stearic acids, along with beta‐sitosterol, cycloeucalenol, cycloartenol, sterol esters, and sterol glycosides (Hussain et al. 2024).

In a meta‐analysis of 16 preclinical studies including a total of 486 experimental animals, Mohammadzadeh et al. investigated the effects of *Nigella sativa* and its constituents on total white blood cell count and airway responsiveness in ovalbumin‐induced asthma models.

The analysis demonstrated that *Nigella sativa* significantly reduced total white blood cells, eosinophils, lymphocytes, and neutrophils (Mohammadzadeh et al. 2025). In another meta‐analysis study, treatment with *Nigella sativa* extract and its active constituents was found to reduce interleukin levels, particularly IL‐4, IL‐5, IL‐13, and IL‐17 (Ghobadi et al. 2025). In a clinical study, 28 children and adolescents with asthma aged 6–15 years were enrolled. Fourteen participants received standard therapy for 8 weeks, while the other 14 were administered 15–30 mL/kg of *Nigella sativa* oil. At the end of the eight‐week period, the group receiving *Nigella sativa* showed improvement in the IFN‐γ/IL‐4 balance and asthma control (Barlianto et al. 2018). Meta‐analyses of numerous studies and outcomes from clinical trials indicate that *Nigella sativa* is beneficial in alleviating inflammation associated with asthma.

### 3.6 Boswellia serrata

*Boswellia serrata* is a species belonging to the Burseraceae family and is a plant resin that typically grows in dry environments, particularly along forest margins in India.

*Boswellia serrata* is an oily, gum‐like resin and is known in pharmacotherapy and official texts as *Gummi Boswellii* or *Olibanum indicum* (Miran et al. 2022). In the treatment of asthma, *Boswellia serrata* exhibits anti‐inflammatory and immunomodulatory effects. In a study conducted by Yugandhar et al. (2018), the use of LI13109F, a herbal formulation containing extracts of *Boswellia serrata* and *Aegle marmelos* , at a daily dose of 200 mg was shown to improve asthma symptoms, increase serum IFN‐γ levels, and reduce IL‐4 levels. Additionally, it was observed to reduce leukotriene‐mediated inflammation by inhibiting the 5‐lipoxygenase pathway. The formulation also decreased granulocyte infiltration and regulated the Th1/Th2 cytokine balance. No serious adverse effects were reported during the study period.

These findings suggest that LI13109F may be an effective supportive therapy in the management of mild to moderate asthma (Yugandhar et al. 2018).

### 3.7 Inula japonica

*Inula japonica* Thunb. (*Asteraceae*), known as “geumbulcho” in Korea and widely distributed in Korea, Japan, and northern China, has traditionally been used in folk medicine to alleviate respiratory disorders, including asthma, cough, and sputum production (Yang et al. 2021). Potential therapeutic effects have been highlighted, including the alleviation of mast cell–mediated allergic reactions, reduction of inflammation and oxidative stress in lung injury, enhancement of exercise endurance, antidiabetic activity, and anti‐asthmatic effects (Jung et al. 2025). In a study conducted using experimental animals, a compound isolated from Inula japonica was found to reduce mucus production and increase lung capacity in mice with ovalbumin‐induced allergic asthma. Additionally, it was observed to significantly inhibit IL‐1β, IL‐4, IL‐5, IL‐6, and IL‐13 levels in lung tissue and suppress the PI3K/AKT/MEK/ERK signaling pathway in the lungs of the mice (Jung et al. 2023). In another study, the effects of Inula japonica extract on asthma were investigated, and it was found to reduce serum total IgE, IL‐4, IL‐5, and IL‐13 levels, as well as decrease macrophage‐derived chemokines and periostin levels. Additionally, it was determined that the extract suppressed the Janus kinase–signal transducer and activator of transcription (JAK–STAT) signaling pathway (Li, Guo, et al. 2023). Preclinical studies indicate that Inula japonica may have beneficial effects in alleviating asthma symptoms.

### 3.8 Astragalus membranaceus

*Astragalus membranaceus* is a medicinal plant that has been used in the treatment of many diseases. In traditional Chinese medicine, *Astragalus membranaceus* has been used to treat weakness, wounds, anemia, fever, chronic fatigue, loss of appetite, and uterine bleeding.

Used in the treatment of various diseases in many countries such as Europe, China, the United States, Japan, and Korea, *Astragalus membranaceus* contains a wide range of chemical constituents, including triterpenoid saponins, flavonoids, and flavonoid glycosides (Li, Liu, et al. 2022). In a study, the role of an *Astragalus membranaceus* extract standardized to 16% polysaccharides in regulating the immuno‐inflammatory response in vitro and in vivo, as well as its therapeutic effects in asthma, were investigated. The study outcomes demonstrated that the *Astragalus membranaceus* extract improved lung function and reduced IgE and Th2 levels (Li, Liu, et al. 2022). In another randomized controlled study, children with asthma were divided into two groups, with one group continuing pharmacological treatment alone, while the other group received adjunct therapy. The control group was treated with a combination of budesonide and terbutaline, whereas the *Astragalus membranaceus* group (treatment group) received a combination of budesonide, terbutaline, and *Astragalus membranaceus*. After 3 months of treatment, therapeutic outcomes were compared between the two groups. Compared with the control group, the treatment group showed a significant reduction in IgE, IL‐17, and IL‐23 levels, along with increased FoxP3 and TGF‐β levels (Wei et al. 2019).

### 3.9 *Justicia adhatoda* (*Adhatoda vasica*)

*Justicia adhatoda* L., also known as *Adhatoda vasica* , is a plant that has been used for many years in Ayurvedic and Unani medicine (Shamsuddin et al. 2021). *Justicia adhatoda* L., belonging to the Acanthaceae family, is widely distributed in the tropical regions of South and Southeast Asia, including India, Pakistan, Sri Lanka, Malaysia, Singapore, Myanmar, southern China, and Laos (Jayaweera et al. 2024). In traditional medicine, *Justicia adhatoda* L. is used in the treatment of respiratory disorders such as whooping cough, chronic bronchitis, and asthma, as well as for the management of excessive sputum production (Sharma et al. 2018). In rat models of acute and severe (steroid‐resistant) asthma, orally administered aqueous *Adhatoda vasica* extract reduced airway resistance, inflammation, and inflammatory markers including IL‐17A, KC, and HIF‐1α (Gheware et al. 2021). There are a limited number of studies regarding the use of *Justicia adhatoda* L. in the treatment of asthma. Further studies are needed to adequately evaluate its effects.

### 3.10 *Piper nigrum* L.

*Piper nigrum* L., which is frequently found on tables worldwide, is commonly consumed for therapeutic purposes in India and Southeast Asia (Chamoli et al. 2022). It has been used in many traditional medical systems, such as Ayurveda and Unani. In traditional medicine, it has been used for the treatment of various respiratory disorders, including cough, common cold, shortness of breath, and asthma. *Piper nigrum* L. may be beneficial in the management of asthma due to its anti‐inflammatory, anti‐allergic, antioxidant, and immunomodulatory effects (Khandekar et al. 2025). In an ovalbumin (OVA)‐induced asthma rat model, oral administration of *Piper nigrum* L. at a dose of 200 mg/kg was found to reduce airway inflammation and fibrosis (Alateeqi et al. 2024). In another study, the effects of *P. nigrum* ethanol extracts (PNE) on airway inflammation were investigated in an asthmatic mouse model. Oral administration of PNE (200 mg/kg) reduced the accumulation of inflammatory cells, such as eosinophils and neutrophils in bronchoalveolar lavage fluid and mast cells in lung tissue, and regulated cytokine production by Th1, Th2, Th17, and Treg cells (Bui et al. 2017). These findings support the therapeutic effects of *Piper nigrum* L. on asthma. However, further studies are required to elucidate its effects on human health.

Plants such as *Glycyrrhiza glabra* , *Curcuma longa* , *Tylophora indica*, *Adhatoda vasica* , *Nigella sativa* , *Boswellia serrata* , *Inula japonica*, *Astragalus membranaceus*, and *Piper nigrum* L. have been used for therapeutic purposes for many years in traditional medical systems in countries such as China and India. The anti‐inflammatory, bronchodilatory, antioxidant, and immunomodulatory effects of these plants suggest that they may have therapeutic potential in asthma. Studies indicate that these plants are also beneficial in suppressing inflammatory processes involved in the pathophysiology of asthma. Although the level of clinical evidence varies among the plants, their effects are not yet fully understood, particularly due to the limited number of randomized controlled trials. Additionally, the long‐term effects of these plants are not fully understood, and information on standardized doses and formulations is limited, representing a significant limitation. While these plants are promising natural agents for asthma treatment, large‐scale, well‐designed clinical studies are needed to clearly establish their efficacy and safety.

## 4 Mechanisms of Herb–Drug Interactions

The immediate rise in the utilization of HMs, their accessibility alongside therapeutic drugs (i.e., non‐prescription), and their simultaneous use with conventional medications to enhance therapeutic outcomes have resulted in a heightened incidence of HDIs and adverse drug reactions (Choi and Song 2021). In recent years, a notable rise in the use of HMs has been recorded, with approximately 20%–35% of patients receiving conventional treatments concurrently employing herbal products (Awortwe et al. 2019). Despite their growing prevalence, numerous public health issues and safety concerns about the concurrent use of herbal and conventional medicines remain largely unresolved today (Pan et al. 2022; Sharma et al. 2022).

The combined use of HMs with medicinal medications may result in advantageous or unforeseen pharmacokinetic interactions. Appropriate HDIs can enhance therapeutic efficacy and diminish side effects, while inappropriate HDIs may provide contrary outcomes. The China National Adverse Drug Reaction Monitoring Centre issued treatment cautions for clinicians by documenting HDI cases that involve adverse effects and dangers in clinical trials to avert such negative occurrences (Song et al. 2022). Simultaneously, the National Institutes of Health in the United States established the Centre of Excellence for Natural Product‐Drug Interactions Research to evaluate the therapeutic relevance of natural product–drug interactions (Cox et al. 2021).

The simultaneous administration of HMs alongside conventional medications has resulted in alterations in the pharmacokinetic and pharmacodynamic properties of the pharmaceuticals (Jermini et al. 2019). These interactions may diminish the therapeutic efficacy of medications, elevate toxicity, or induce unexpected side outcomes. Moreover, interactions can influence the pharmacological efficacy, serum concentrations, metabolism, or toxicity of allopathic medications. Interactions between HMs and drugs were based on the same pharmacological principles as those governing DDIs. In contrast to allopathic pharmaceutical treatments that consist of a singular molecule, HMs typically comprise a complex amalgamation of phytochemicals generated as secondary metabolites. A HM, such as an extract or plant material, may comprise over 150 components, complicating the identification of side effects and interactions. Moreover, adverse effects resulting from interactions with HM were typically underreported and inadequately recorded, and patients' unwillingness to disclose their usage to healthcare providers further complicates the issue (Gouws and Hamman 2020). HDIs primarily influence metabolic enzymes, regulate carrier proteins, and exhibit pharmacodynamic synergy or antagonism (Cheng et al. 2023). However, difficulties remain in this domain, primarily because most studies possess insufficient understanding of the variety of plant compounds, the ambiguities associated with pathogenic components, and the pharmacokinetic mechanisms of active plant constituents. Challenges and complexities in thoroughly assessing HDIs endure.

### 4.1 Pharmacokinetic Mechanisms of Herb–Drug Interactions

The pharmacokinetic principles behind plant–drug interactions have been clarified by the manner in which herbal products influence the absorption, distribution, metabolism, and excretion (ADME) of pharmaceuticals, resulting in clinically relevant alterations in systemic drug exposure. Current evidence indicates that these interactions predominantly occur through metabolic enzymes and transporter proteins; specifically, the inhibition or induction of cytochrome P450 (CYP450) isoenzymes and membrane transporters like P‐glycoprotein (P‐gp) substantially influence the pharmacokinetic profiles of concurrently administered drugs (Cheng et al. 2023). Flavonoids, alkaloids, and other bioactive constituents in plant products have been shown to modify enzyme expression and transporter activity in essential ADME organelles, such as the gut and liver, thereby affecting the bioavailability, plasma concentration, and clearance rate of pharmaceuticals (Czigle et al. 2023). Reportedly, these pharmacokinetic alterations may result in diminished therapeutic efficiency in certain instances and heightened toxicity risk in others; thus, it is crucial to meticulously assess plant‐drug interactions for safe and sensible therapy (Cheng et al. 2023; Pan et al. 2022). Moreover, the gut microbiota's metabolism of plant constituents and its impact on the production of active or inactive metabolites has been recognized as an additional pharmacokinetic mechanism influencing the severity of plant‐drug interactions, demonstrating clinical significance, especially in multi‐component herbal formulations (Liang et al. 2022). The absorption of drugs in the gastrointestinal system is the initial and paramount phase of the ADME process, during which plant‐drug interactions may occur.

#### 4.1.1 Modulation of Drug Absorption in the Gastrointestinal Tract

The primary method of administering HMs has been oral, designating the gastrointestinal tract as the main location for medication absorption and presystemic metabolism. Compounds produced from plants have been shown to influence the permeability of intestinal epithelial membranes and regulate essential metabolic enzymes and transport proteins, thus modifying the absorption kinetics of concurrently delivered medications. Piperine represents a well‐defined example, as it enhances systemic drug exposure by inhibiting intestinal P‐glycoprotein (P‐gp) and CYP3A4 activity, directly affecting the absorption of drugs and first‐pass metabolism (Gidwani et al. 2025). Conversely, some phytochemicals were documented to diminish drug absorption by creating insoluble complexes or modifying luminal physicochemical conditions, resulting in reduced solubility and compromised transporter‐mediated uptake. Moreover, it has been observed that natural compounds, termed bioenhancers, enhance intestinal permeability via components such as saponins, flavonoids, and alkaloids, modify the expression of carrier proteins, and consequently influence the absorption kinetics of medications (Czigle et al. 2023; Gidwani et al. 2025; Li, Du, et al. 2022). All of these results point to the gastrointestinal system as a crucial and ever‐changing site for pharmacokinetic herb–drug interactions, which directly affects therapeutic effectiveness, dosage optimization, and the likelihood of negative drug reactions in clinical settings.

#### 4.1.2 Enzymes Involved in Drug Metabolism in Herb–Drug Interactions: Cytochrome P450 and Phase II Conjugative Enzymes

Drug‐metabolizing enzymes were essential for transforming medicines into metabolites with distinct physicochemical and biological characteristics (Lippert and Renner 2022). There were two types of in vivo drug metabolism: Phase I and Phase II reactions. Phase I processes encompassed oxidation, reduction, and hydrolysis, incorporating reactive or polar functional groups (—OH, —COOH, —NH_2_, —SH) into the molecules of drugs, which typically remained inadequate for direct excretion. Phase II reactions eventually associated these changed compounds with polar molecules using transferase enzymes, mainly UDP‐glucuronosyltransferases (UGTs), SULTs, and glutathione S‐transferases (GSTs) (Zhao et al. 2021). While conjugation typically aids in detoxification and excretion via efflux transporters, analogous metabolic pathways may also produce reactive intermediates, a phenomenon dubbed drug bioactivation, contingent upon particular structural characteristics of the parent chemical (Song et al. 2021).

The metabolism of drugs mediated by CYP enzymes is essential in influencing treatment outcomes, including drug efficacy, safety, bioavailability, and resistance (Zhao et al. 2021). These enzymes were mostly found in hepatic microsomes, with secondary expression in the gut, lung, kidney, and brain. Of the 57 functional human CYP isoforms, the CYP1, CYP2, and CYP3 families jointly enable almost 80% of clinical drug metabolism, with CYP3A4 and CYP2D6 responsible for over half of all CYP‐dependent responses. Personal variation in CYP‐mediated metabolism is influenced by genetic and epigenetic variables, environmental factors such as age, sex, nutrition, and illness states, and is additionally affected by enzyme inhibition or induction from concurrent use of drugs or HMs (Hossam Abdelmonem et al. 2024; Mokhosoev et al. 2024).

CYP3A4, the predominant Phase I enzyme in vivo, constituted 30%–40% of total hepatic CYP450 content and mediated the metabolism of approximately 50% of administered pharmaceuticals (Vander Schaaf et al. 2024). Due to its broad substrate selectivity and significant vulnerability to manipulation, CYP3A4 has emerged as the most thoroughly studied isoenzyme regarding drug–drug and herb–drug interactions. Different HMs have been documented to stimulate CYP3A4 expression, while others demonstrated inhibitory actions, leading to clinically significant changes in systemic drug exposure (Czigle et al. 2023; Denison et al. 2023). Genetic factors additionally influenced variability in CYP function, with over 350 functional CYP alleles recognized in humans (Zhou et al. 2017). Polymorphisms in CYP2D6, CYP2B6, and CYP2A6 shown significant allelic diversity, considerably influencing drug clearance. Loss‐of‐function mutations diminished metabolic clearance and elevated plasma drug levels, while gain‐of‐function variants enhanced metabolism and decreased systemic exposure, therefore altering both therapeutic effectiveness and toxicological risk (Frederiksen 2023; Langmia et al. 2021).

UGTs are the principal hepatic enzymes in Phase II metabolic processes, facilitating the biotransformation of many medicines by UDP‐glucuronic acid (UDPGA)‐mediated glucuronidation (Mano et al. 2018). The UGT superfamily has four primary groups, with the UGT1 and UGT2 subfamilies identified as the principal contributors to drug glucuronidation, accounting for roughly 35% of Phase II drug metabolism (Abdullah and Ismail 2018). Highly selective substrates and inhibitors for UGTs have been sourced from various origins, including natural products; specifically, resveratrol has been demonstrated to enhance UGT1A8 expression and was utilized clinically in breast cancer therapy, whereas emodin, for example, was shown to inhibit UGT2B7 activity in a dose‐dependent way. Multiple UGT isoforms have been discovered to metabolize plant compounds in some instances. Linoleic acid and glutaric acid documented to impede the glucuronidation of berberrubin, a lipid‐reducing metabolite of berberine, as well as the activities of isoforms including UGT1A7, UGT1A8, and UGT1A9. Research indicated that the glucuronidation of catalpozide, the active constituent of Veronica species, is facilitated by the gastrointestinal‐specific enzymes UGT1A8 and UGT1A10 (Liu et al. 2023; Meng et al. 2021; Nasrin et al. 2021).

Genetic polymorphisms are critical factors in the modulation of UGT expression and function (Meng et al. 2021). Polymorphisms in the UGT1A and UGT2B genes were documented to modify the glucuronidation of nicotine and nitrosamines in European and African American smokers. Moreover, research indicated that the UGT1A4*3 polymorphism is linked to reduced posaconazole plasma levels in individuals with malignancies of the blood, and the UGT1A1*6 polymorphism is connected with irinotecan‐related neutropenia in patients with cancer. It has recently been established that UGT1A3 was involved in the glucuronidation of alpinetin, whereas UGT1A4 was implicated in the glucuronidation of metizolam (Atasilp et al. 2022; Geng et al. 2025; Nelson et al. 2021).

It has been determined that SULTs and GSTs are important conjugation enzymes that mediate Phase II metabolic events in addition to UGTs (Isvoran et al. 2022; Mazari et al. 2023). SULTs facilitate the transfer of the water‐soluble sulfate moiety from 3′‐phosphoadenosine‐5′‐phosphosulfate to pharmaceuticals or endogenous compounds possessing hydroxyl or amine functionalities. SULT1, SULT2, SULT4, and SULT6 are the four families that include human cytosolic SULTs, which comprise a total of 12 enzymes present in various tissues (Huang et al. 2022). SULT1E1 is essential for the synthesis and detoxifying of estrogens and flavonoids. SULT1E1 is essential for the synthesis and detoxifying of estrogens and flavonoids. SULTs participate in the metabolism of several substrates, encompassing endogenous chemicals such catecholamines and steroids, in addition to natural products and medicines. Cytosolic SULTs were demonstrated to be therapeutically significantly inhibited by nonsteroidal anti‐inflammatory medications (NSAIDs), and molecular processes behind drug interactions involving several SULT isoforms have been clarified. Moreover, it has been underscored that various genetic variants associated with each SULT isoform may significantly influence inter‐individual variability in medication responsiveness and toxicity, as well as elevate the risk of specific disorders (Isvoran et al. 2022; Xie and Xie 2020). GSTs are essential detoxifying enzymes that play a critical role in Phase II drug metabolism by catalyzing the conjugation of decreased glutathione (GSH) to diverse electrophilic molecules. These enzymes are essential for neutralization reactive intermediate metabolites and mitigating cellular oxidative stress. Cytosolic GST isoenzymes found in humans have been classified into alpha, zeta, theta, mu, pi, sigma, and omega classes, exhibiting notable variations in tissue distribution and substrate specificity. Evidence indicated that the GSTA1 isoenzyme was crucial for the elimination of toxic metabolites of acetaminophen, whereas GSTA4 was implicated in the breakdown of electrophilic molecules like 4‐hydroxy‐2‐nonenal, recognized as a reactive product of peroxidation of lipids and an endogenous cancer‐causing agent. These roles of GST isoenzymes have prevented the buildup of medication‐induced reactive substances and endogenous hazardous chemicals at the cellular level, hence limiting tissue damage. Moreover, it was highlighted that individual variations in GST activity may significantly influence the variability in medication reaction and vulnerability to toxicity (Lv et al. 2023; Mazari et al. 2023; Stoian et al. 2025).

The combined modulation of Phase I and Phase II drug metabolism enzymes by genetic variation, herbal products, and combined pharmaceutical therapy has become a crucial factor in pharmacokinetic HDIs and DDIs, highlighting the importance of including enzymatic regulation in risk identification, dose optimizing, and personalized treatment strategies.

#### 4.1.3 Pharmacokinetic Herb–Drug Interactions: Function of Drug Transporters

Transport proteins are membrane‐associated structures present in several organs, enabling the absorption or transport of various endogenous and exogenous substances, including medicines and metabolites of them, into and out of cells (Li et al. 2019). Transporters serve as critical “gatekeepers” in pharmacotherapy, regulating cellular uptake and efflux mechanisms, and so significantly impacting the overall ADME process. Changes in carrier activity or regulatory expression can significantly impact blood and tissue exposure to drugs, resulting in notable variations in pharmacokinetic characteristics and ultimately affecting the balance between therapeutic efficacy and toxicity (Li, Yin, et al. 2023; Trucillo 2022).

Uptake carriers are membrane‐bound proteins that are part of the solute carrier (SLC) superfamily, specifically including organic anion‐transporting polypeptides (OATPs), organic anion transporters (OATs), and organic cation transporters (OCTs) (Brecht et al. 2020). These transporters are highly prevalent in organs essential for drug pharmacokinetics, including the liver, kidneys, and gastrointestinal tract. The primary function of uptake transporters is to regulate the transport of small‐molecule drugs and endogenous substrates into cells, hence affecting the absorption in the gastrointestinal tract, hepatic uptake (first‐pass metabolism), tissue distribution, and renal excretion of pharmaceuticals. The expression and function of these transporters can be altered by concurrently administered pharmaceuticals and herbal products, leading to significant variations in systemic exposure, tissue distribution, and clinical response, thus contributing to clinically significant HDIs and DDIs (Brouwer et al. 2022; Järvinen et al. 2022).

Efflux transporters are transmembrane proteins that are part of the ATP‐binding cassette (ABC) transporter superfamily, which includes P‐glycoprotein (P‐gp), breast cancer resistance protein (BCRP), and multidrug resistance proteins (MRPs) (Li, Wang, et al. 2022). These transporters are prominently present in the outer membranes of glandular organs, including cells of the intestine, hepatocytes, and kidney tubule cells, as well as in certain physiological barriers such as the blood–brain barrier and blood‐testis barrier. Efflux transporters primarily function to enable the ATP‐dependent active extrusion of drugs and other xenobiotics from the cell to the intestines, biliary system, or urine tract. This process reduces intracellular accumulation of potentially toxic substances, hence reducing systemic exposure and significantly influencing bioavailability, particular organ distribution, and response to treatment (Eng et al. 2022; Remião 2020). Transporter‐mediated regulation, along with drug‐metabolizing enzymes, constitutes a fundamental factor in pharmacokinetic variability and serves as a critical mechanistic foundation for HDIs and DDIs, highlighting the necessity of comprehensive transporter‐enzyme evaluation in clinical risk management and personalized pharmaceutical therapy.

### 4.2 Pharmacodynamic Mechanisms of Herb–Drug Interactions

Pharmacodynamic interactions refer to the alterations in the effects of multiple substances, such as medications or physiologically active herbal substances, at their target locations (e.g., receptors, enzymes, or ion channels) or through associated physiological systems (Czigle et al. 2023). These interactions may arise from direct competition between drugs for identical objectives, but more frequently they occur via indirect modification of signaling pathways in cells or biological processes. When agents exhibiting comparable or identical pharmacological effects are co‐administered, additive or synergistic effects may manifest, enhancing therapeutic efficacy and, in certain instances, diminishing toxicity; conversely, the concurrent use of compounds with opposing effects may result in antagonistic interactions, thereby decreasing drug efficacy or heightening the risk of adverse effects (Chaachouay 2025).

The basic mechanisms of pharmacodynamic interactions primarily take place through receptors, enzymes, ion channels, and various cellular targets; within these processes, biologically active compounds function as agonists, antagonists, enzyme inhibitors, or modulators (Narwal et al. 2025). In the current research, documented interactions between *Ginkgo biloba* and antithrombotic agents (Mai et al. 2025), as well as between *Hypericum perforatum* and antidepressants, illustrate instances of clinically important pharmacodynamic effects (Surana et al. 2021). It is highlighted that patient safety could be at risk when there is a lack of information regarding potential interactions between plants and drugs. However, it is also noted that certain pharmacodynamic interactions, when thoroughly assessed, can provide avenues for the thoughtful development of combination therapies, enhancing treatment effectiveness and paving the way for novel therapeutic approaches in the future (Niu et al. 2019).

Pharmacodynamic HDIs occur when bioactive compounds from plants and conventional drugs modify each other's effects at the site of action, such as receptors, enzymes, or ion channels, or along the same physiological pathway. Clinical outcomes are primarily influenced by the nature of these interactions, which can be characterized as synergistic, additive, or antagonistic. Recent study highlights that pharmacodynamic interactions generally modify the pharmacological response “without altering the drug concentration”; this is achieved through receptor‐level agonism or antagonism, functional inhibition at the enzyme level, or modulation of cellular pathways (such as platelet function, serotonergic tone, and inflammatory response) (Czigle et al. 2023; Hossain et al. 2022). Clinical reports indicate that the concurrent application of two agents focusing on the same biological target or endpoint may enhance their effects either additively or potentiatingly. In contrast, combinations that act in opposing directions can diminish the therapeutic response, resulting in reduced effectiveness and an increase in adverse effects (Duan et al. 2025; Puthiyedath and Pillai 2025). This mechanistic framework demonstrates that pharmacodynamic interactions correlate with clinical safety outcomes in both theoretical and practical settings.

Pharmacodynamic synergies and additiveness are increasingly evident in physiological processes that are sensitive to reduced safety limits, such as hemostasis and brain signaling. It has been contended that certain herbal products, when utilized alongside antithrombotic or anticoagulant medications, may elevate the danger of hemorrhage due to their influence on platelet function or coagulation factors, and that this risk possesses a pharmacodynamic aspect (Mai et al. 2025; Talasaz et al. 2024). In the serotonergic system, it was observed that herbal products may enhance serotonergic activity or functionally coincide with serotonergic medications, establishing a pharmacodynamic interaction that could result in negative consequences such as serotonin toxicity or serotonin syndrome. Although the evidence is frequently derived from case reports and theoretical consistency, it is regarded as a “high‐risk scenario” clinically (Blumenfeld et al. 2023; Spanakis et al. 2025). Recent extensive analyses have demonstrated that herbal‐drug combinations may offer therapeutic benefits; however, the same mechanisms can also result in antagonism or heightened toxicity. Consequently, pharmacodynamic HDIs should be regarded with equal clinical severity as DDIs, necessitating a target/pathway‐based risk evaluation (Déciga‐Campos et al. 2022; Puthiyedath and Pillai 2025).

Integrated pharmacokinetic–pharmacodynamic HDIs gain clinical importance as pharmacokinetic modifications, initiated by herbal products altering systemic drug exposure (AUC, Cmax) through metabolic enzymes and transport proteins, redefine the efficacy‐toxicity equilibrium within the therapeutic period and target tissue response. Pharmacokinetic alterations due to the modulation of CYP enzymes or drug transporters may coincide with synergistic or antagonistic effects at the pharmacodynamic level, heightening the risk of adverse effects; nevertheless, in certain combinations, it may be feasible to optimize exposure and target response in a regulated manner. Recent recommendations and modeling approaches, especially through physiologically based pharmacokinetic models, have sought to mechanistically predict integrated pharmacokinetic–pharmacodynamic interactions and translate this into clinical risk management, offering a more systematic and predictable framework for assessing HDIs (Taş and Ulgen 2025; Yang et al. 2025).

## 5 Potential Interactions Between Herbal Medicines and Anti‐Asthma Drugs

A considerable percentage of drugs utilized in anti‐asthma therapy, especially specific ICS and certain LABA, are metabolized through CYP enzymes and/or transporter proteins. The induction or inhibition of these systems by HMs has resulted in clinically relevant alterations in drug exposure. Inhaled and intranasal corticosteroids are metabolized through CYP3A4 pathways. The concurrent use of strong CYP3A4 inhibitors can elevate systemic corticosteroid exposure, potentially resulting in conditions such as Cushing's syndrome or adrenal suppression. This indicates that herbal CYP3A4 inhibitors, which exert a comparable effect, may also present a risk (Carr et al. 2023). The FDA pointed out that dietary or plant‐derived CYP3A4 inhibition, exemplified by grapefruit constituents, may elevate systemic exposure by diminishing intestine first‐pass metabolism (Grams et al. 2025). Herbal products like St. John's wort ( *Hypericum perforatum* ), which are strong inducers of enzymes and transporters, can diminish the bioavailability and effectiveness of various medications; consequently, they may elevate the risk of clinical failure in long‐term maintenance therapies, especially for drugs with a limited therapeutic window or those sensitive to exposure (Nicolussi et al. 2020).

At the drug class level, disparities in metabolic pathways among LABAs have influenced the risk of interactions: for instance, salmeterol is metabolized by CYP3A4, while certain LABAs (e.g., formoterol) are not dependent on CYP3A4 and are consequently not anticipated to exhibit the same degree of interaction risk with potent CYP3A4 inhibitors. Recent reviews have extensively examined the metabolism and safety profile of montelukast, LTRA. It has been argued that although montelukast demonstrates significant in vitro inhibition of CYP2C8, this may not consistently result in clinical interactions due to factors like high protein binding (Marques et al. 2022; Zhu et al. 2025). However, data was emerged about the assessment of the simultaneous use of herbal products and montelukast concerning safety signals, indicating that concurrent use with specific HMs may necessitate caution due to potential hepatic adverse effects (Di Giacomo et al. 2023). As a result, there exists a potential for interaction when evaluating anti‐asthma medications alongside HMs. This assessment considers the drug's dependence on CYP and transporters, the herbal product's potential for induction or inhibition, the dosage and duration of use, as well as patient‐specific factors such as comorbidities and the use of multiple medications (Li, Jia, et al. 2022). This highlights the importance of adopting a mechanism‐based strategy in managing risks associated with HDI/DDI (Bukowska et al. 2025; Liang et al. 2022).

Table 3 illustrates that HMs can both augment and interact with conventional drugs in asthma treatment due to their anti‐inflammatory, bronchodilator, and immunomodulatory properties. To safely incorporate these potential benefits into clinical practice, standardized preparations, pharmacokinetic–pharmacodynamic interaction assessments, and high‐quality randomized controlled trials are essential. Diverse HMs and their bioactive constituents demonstrated impacts on inflammatory, immunological, and bronchoconstrictive pathways implicated in the pathogenesis of asthma via multiple targets. HMs including *Glycyrrhiza glabra* , *Curcuma longa* , *Nigella sativa* , *Boswellia serrata* , *Adhatoda vasica* , and *Tylophora indica*; attenuation of Th2 cytokines (IL‐4, IL‐5, IL‐13), inhibition of NF‐κB and MAPK signaling pathways, mitigation of eosinophilic inflammation, and normalization of airway reactivity (Boskabady et al. 2021; Ghobadi et al. 2025; Naufal et al. 2021; Qi et al. 2023; Sarma 2020; Soni et al. 2020). It indicates that the plants may demonstrate corticosteroid‐like or corticosteroid‐enhancing actions.

**TABLE 3:** Interactions of pharmacological agents in asthma treatment with herbal medicines.

Plant	Drug class	Key mechanism	Interactions with anti‐asthma drugs
*Glycyrrhiza glabra* (Nandhini et al. 2023; Fouladi et al. 2019; Qi et al. 2023)	Inhaled/systemic corticosteroids	Licorice components (glycyrrhizin, glycyrrhizic acid, licochalcone A, glabridin) inhibited NF‐κB, Th2 cytokines (IL‐4, IL‐5, IL‐13), eosinophils, and IgE, while augmenting IFN‐γ and Tregs, similarly to the anti‐inflammatory effects of glucocorticoids	Possible additive or synergistic anti‐inflammatory impact; however, there is a danger of increased steroid‐like effects due to 11β‐HSD2 inhibition and potential pharmacological interactions with corticosteroids
	Leukotriene modifiers	Licorice flavonoids and glycyrrhizic acid mitigate eosinophilic lung inflammation and subsequent mediators influenced by Th2 cytokines and NF‐κB activation	May enhance leukotriene antagonists through upstream inhibition of Th2 and NF‐κB pathways implicated in leukotriene synthesis and eosinophilia
	β_2_‐Agonists (SABA/LABA)	Glycyrrhizin exhibited synergistic anti‐asthmatic effects with salbutamol in silico; nano‐assemblies can co‐deliver salbutamol and glycyrrhizin to inflamed airways	Potential synergy with bronchodilators, enhancing targeting and anti‐inflammatory regulation; does not function as a conventional bronchodilator
	Anti‐Th2 therapies	Glycyrrhizin and glycyrrhizic acid decrease IL‐4, IL‐5, IL‐13, TNF‐α, IL‐17, CXCL1/2, while augmenting IFN‐γ and Tregs in OVA‐asthma mice	There are overlaps with Th2‐ and IL‐5/IL‐13‐targeted biologics, indicating that licorice may offer a more extensive and cost‐effective upstream immune regulation, while only preclinical evidence is available
	General anti‐inflammatory	Whole plants and their constituents (glycyrrhizin, glycyrrhizic acid, glabridin) consistently demonstrated a reduction in airway hyperresponsiveness, eosinophilia, mucus production, and oxidative stress, along with superior outcomes compared to steroids in animal models of asthma	Supporters for the utilization of *Glycyrrhiza glabra* as a supplementary nutraceutical/phyto‐medicine in asthma; nevertheless, clinical trials are few, and long‐term safety alongside potential drug–drug interactions, particularly with corticosteroids, warrants careful consideration
*Curcuma longa* (Boskabady et al. 2021; Naufal et al. 2021)	Inhaled/systemic corticosteroids	Substantial suppression of NF‐κB and MAPK (p38, ERK, JNK), decreased levels of COX‐2, PLA_2_, Th2 cytokines (IL‐4, IL‐5, IL‐13), IgE, eosinophils, and airway remodeling; effects equivalent to or exceeding those of dexamethasone in animal models	Mechanistically parallels corticosteroids as an upstream anti‐inflammatory and anti‐remodeling agent; indicates potential as a dose‐sparing adjuvant, however clinical evidence in humans remains limited and inconsistent
	β_2_‐Agonists (SABA/LABA)	Enhanced airway hyper‐responsiveness and blockage, diminished mucus and smooth muscle hypertrophy; oral *Curcuma longa* in children decreased the necessity for short‐acting β_2_‐agonists and enhanced control metrics	Primarily functions as an anti‐inflammatory agent/controller rather than a direct bronchodilator; may reduce the necessity for rescue β_2_‐agonists when incorporated into conventional treatment
	Leukotriene modifiers	Reduced PLA_2_ activity, prostaglandin D_2_ release, Th2 cytokines, eotaxin, and oxidative stress, thereby diminishing eosinophilic inflammation and AHR	Facilitates upstream inhibition of eicosanoid and Th2‐mediated pathways, potentially augmenting leukotriene receptor antagonists as an adjunct anti‐inflammatory therapy
	Biologics targeting Th2/IgE (anti‐IL‐4/5/13, anti‐IgE)	Downregulated IL‐4, IL‐5, IL‐13, TNF‐α, TGF‐β, eotaxin, and OVA‐IgE; changes Th1/Th2 equilibrium, elevated IFN‐γ and regulatory/anti‐inflammatory responses, and affected Notch1/2‐GATA3 and IL‐4Rα‐JAK1‐STAT6 pathways	Imitates extensive upstream Th2‐pathway and IgE regulation at many points; may provide a cost‐effective adjunct or alternative in environments lacking biologics, while existing data are either preclinical or derived from limited trials
	Anti‐remodeling/add‐on controller therapy	Intranasal and oral curcumin, together with *Curcuma longa* extract, mitigate airway remodeling (including smooth muscle and wall thickening, collagen, MMP‐9, VEGFA), mucus hypersecretion, and oxidative stress; certain formulations (liposomes, intranasal) surpass conventional medications in experimental animals	Curcuma/curcumin (together with curcumol and tetrahydrocurcumin) is supported as a viable adjunctive treatment for inflammation and remodeling; nevertheless, formulation and bioavailability present significant obstacles to clinical application
*Tylophora indica* (Gururani et al. 2023; Khurana 2019)	Inhaled/systemic corticosteroids	Extracts diminished airway hyperresponsiveness, bronchoalveolar lavage fluid IgE and IL‐4/IL‐13 levels, as well as inflammatory cytokines in OVA‐asthma guinea pigs; phenanthroindolizidine alkaloids (e.g., tylophorine) exhibited potent anti‐inflammatory and immunomodulatory effects	Offers anti‐inflammatory and immunomodulatory regulation that partially coincides with steroids; may function as an auxiliary controller but does not supplant the substantial evidence supporting corticosteroids
	β_2_‐Agonists (SABA/LABA)	The chloroform leaf fraction induced relaxation of guinea pig tracheal rings by β_2_‐adrenergic stimulation, activation of soluble guanylyl cyclase, and inhibition of contraction pathways (MLCK/ROCK)	Exhibits genuine bronchorelaxant properties; may enhance β_2_‐agonists or partially replace them as a bronchodilator in herbal preparations, however the evidence remains preclinical
	Antihistamines (H1 blockers)	The chloroform leaf fraction exhibited non‐competitive antagonism at histamine receptors in tracheal smooth muscle; intranasal extract provided protection against histamine‐induced bronchoconstriction in guinea pigs	Mechanistically coincides with H1‐antihistamines, indicating a potential decrease in histamine‐mediated bronchospasm and potentially dose‐sparing when used in combination, subject to clinical validation
	Mast‐cell stabilizers/anti‐allergic agents	Traditional and clinical studied indicate advantages in allergic asthma and rhinitis; preliminary trials utilizing leaf or alcoholic extract demonstrated symptom alleviation for 8–12 weeks following a brief 6‐day regimen, presumably through anti‐allergic and immunomodulatory mechanisms	May serve as a long‐acting anti‐allergic adjuvant, potentially diminishing attack frequency and the necessity for other medications, however side effects (nausea, sore mouth) and inconsistent results have been seen
	General controller/phytomedicine	Established Ayurvedic application for bronchial asthma; clinical trials, intranasal administration, and animal studies demonstrated antiasthmatic effectiveness (bronchodilation, diminished hyperreactivity, enhanced symptoms); primary active constituents were phenanthroindolizidine alkaloids (tylophorine, tylophorinidine) exhibiting anti‐inflammatory and immunomodulatory properties	*Tylophora indica* is endorsed as a multi‐target antiasthmatic phytomedicine, exhibiting bronchodilator, antihistaminic, anticholinergic, and anti‐inflammatory properties; however, its interactions with conventional medications and long‐term safety necessitate thorough investigation
*Adhatoda vasica* (Gheware et al. 2021; Gujjarwar 2025; Peron et al. 2024; Sarma 2020)	Inhaled/systemic corticosteroids	Methanolic/aqueous leaf extracts exhibited a more potent inhibition of NF‐κB and AP‐1 than prednisolone in vitro, and demonstrate anti‐inflammatory, antitussive, and anti‐asthmatic effects in animal models. Additionally reduced IL‐17A, KC, HIF‐1α, and reinstates PHD2 in severe, steroid‐resistant asthma animals	Exhibits extensive anti‐inflammatory and steroid‐sparing capabilities, particularly in hypoxia‐driven, steroid‐resistant asthma; nevertheless, clinical evidence in humans remains insufficient
	β_2_‐Agonists (SABA/LABA)	Vasicine and vasicinone exhibited bronchodilatory actions both in vitro and in vivo, demonstrating efficacy comparable to theophylline in animal models; leaf extracts prolong pre‐convulsion duration in histamine/acetylcholine‐induced bronchoconstriction in guinea pigs	Functions as a true bronchodilator; may enhance or partially replace low‐dose β_2_‐agonists in herbal formulations, but should not supplant fast‐acting inhaled SABA in the absence of clinical trials
	Biologics targeting Th2 cytokines (IL‐4/IL‐13)	Docking and analytical analyses demonstrated that several quinazoline alkaloids (such as vasicoline/vasicolinone and vasicinol) interact with IL‐4 and IL‐13 with advantageous binding energies, suggesting direct targeting of the Th2 pathway	Indicates a multi‐target, upstream regulation of Th2 cytokines; may theoretically enhance biologics, while evidence remains solely preclinical
*Nigella sativa* (Areej et al. 2025; Barlianto et al. 2018; Ghobadi et al. 2025; Gholamnezhad et al. 2019; He and Xu 2019)	Inhaled/systemic corticosteroids	Strong anti‐inflammatory, antioxidant, and immunomodulatory effects: diminished Th2 cytokines (IL‐4, IL‐5, IL‐13, IL‐17) and IgE in OVA‐asthma models; regulated NF‐κB and other inflammatory pathways	Acts as a multi‐target controller agent that may exhibit steroid‐sparing effects; evidence endorses its usage as an adjunct to regular ICS rather than a replacement
	β_2_‐Agonists (SABA/LABA)	Boiled seed extract induced rapid bronchodilation in asthmatic patients; its onset and persistence were comparable to theophylline, albeit with a little lesser effect than both theophylline and salbutamol on several flow metrics	Functions as an actual bronchodilator; may be utilized as an oral adjunct bronchodilator, but not as a substitute for rapid‐onset inhaled β_2_‐agonists
	Biologics/Th1‐Th2; Th17‐Treg modulators	*Nigella sativa* and thymoquinone altered the Th1/Th2 balance (↑IFN‐γ, ↓IL‐4) and enhanced the Th17/Treg ratio in children; a preclinical meta‐analysis corroborated extensive Th2‐cytokine suppression	Proposes upstream immunologic regulation akin to biologics that target Th2/Th17 pathways, albeit with reduced selectivity and primarily preclinical or early clinical evidence
	Standard controller therapy	Numerous randomized controlled trials and meta‐analyses demonstrated enhanced ACT scores, FEV_1_, FEF25‐75, PEF variability, FeNO, IgE levels, and eosinophil counts when nebulized saline was incorporated into standard asthma therapy for both adults and children	*Nigella sativa* (seeds, oil, or extract) is advocated as a valuable complement to traditional anti‐asthma therapies, potentially enhancing management and decreasing reliance on rescue or maintenance medications; standardized formulations and extended, larger‐scale studies are necessary
*Boswellia serrata* (Gomaa et al. 2021; Hewedy 2020; Soni et al. 2020)	Inhaled/systemic corticosteroids	Extracts downregulated Th2 cytokines (down IL‐4, increase IFN‐γ), diminish eosinophils, lung leukocytes, and methacholine hyperreactivity in animal models of asthma; modifyed NF‐κB, COX‐2, and immunological responses	Functions as a multi‐pathway anti‐inflammatory agent, potentially reducing the need for steroids; nonetheless, inhaled corticosteroids cannot be substituted according to existing evidence
	β_2_‐Agonists (SABA/LABA)	The extract of *Boswellia serrata* induced relaxation of pre‐contracted rat trachea in a concentration‐dependent manner and enhanced isoprenaline‐induced relaxation, irrespective of the epithelium or nitric oxide/prostaglandins	Gives direct bronchodilation and may augment the efficacy of β_2_‐agonists, indicating potential utility as an adjuvant; nevertheless, evidence is inadequate to support its use as a sole rescue bronchodilator
	Overall clinical controller	Randomized controlled trials with *Boswellia serrata* gum resin (300 mg three times daily for 6 weeks) and a standardized combination with *Aegle marmelos* (AlvioLife/LI13109F) indicate enhanced symptoms, lung function (FEV_1_, PEFR), and reduced eosinophil levels in mild to moderate asthma, demonstrating good tolerability	Supports *Boswellia serrata* as a controller‐type adjuvant or nutraceutical for mild to moderate asthma; nevertheless, dose, standardization, and interactions with conventional therapy require further elucidation
*Inula japonica* (Li, Guo, et al. 2023)	Inhaled corticosteroids	Water or ethanol extracts diminished AHR, eosinophil infiltration, Th2 cytokines (IL‐4, IL‐5, IL‐13), eotaxin, periostin, mucus hypersecretion, collagen deposition, and total/OVA‐IgE levels in OVA‐asthma mice	Functions as a multi‐pathway anti‐inflammatory agent, potentially reducing the need for steroids; nevertheless, the data are derived from animal and cellular studies and cannot substitute for ICS
*Astragalus membranaceus* (Bunddulam et al. 2025; D'Avino et al. 2023; Yuan et al. 2023)	Inhaled corticosteroids	Root extract diminished PGE_2_, leukotriene C_4_, NO, TNF‐α, IL‐1β, IgE, Th2 cytokines (IL‐4, IL‐5, IL‐13), AHR, airway remodeling, and fibrosis in OVA‐asthma mice. Astragalus polysaccharides mitigated severe eosinophilic‐neutrophilic inflammation, endoplasmic reticulum stress, NF‐κB activation, and mucus production (MUC5AC/5B)	Operates as a multi‐pathway anti‐inflammatory regulator; may reduce the need for steroids but cannot substitute for inhaled corticosteroids based on current research
	Leukotriene‐pathway	Extract and leaf extract suppressed leukotrienes (LTC_4_, LTB_4_), histamine release, 5‐LOX activity, Ca^2+^ influx, and NF‐κB in mast cells and macrophages	Partial crossover with leukotriene antagonists and mast cell stabilizers; possible adjuvant to mitigate early‐phase allergy responses
	Airway remodeling	Astragaloside‐IV inhibited airway smooth muscle cell proliferation, promotes pyroptosis through the HMGB1/RAGE‐NF‐κB pathway, and diminished remodeling and inflammation; APS mitigated remodeling associated with endoplasmic reticulum stress; the total extract restricts fibrosis	Important to remodeling‐oriented therapy in chronic/severe asthma; remains experimental
*Piper nigrum* (Christianty et al. 2024; Duan et al. 2022; Gulati 2022; Khawas et al. 2017)	Controller	*Piper nigrum* decreased airway hyper‐responsiveness, OVA‐IgE, and TNF‐α levels in blood and BALF in OVA‐asthma rats, comparable to prednisolone. PNE in OVA‐asthma mice reduced eosinophils, neutrophils, mast cells, Th2/Th17 cytokines, TNF‐α, and fibrosis, while elevating IL‐10 and IFN‐γ	Controller‐like anti‐inflammatory/immunomodulator; potential supplementary treatment to ICS, but not a substitute
	Respiratory symptom relief	The aqueous extract of *Piper nigrum* , enriched with pectic polysaccharides and piperine, exhibited antitussive effect comparable to codeine in guinea pigs; individual fractions shown diminished efficacy, although their combination proved synergistic without impacting airway smooth muscle reactivity	Symptomatic cough suppressant that complements bronchodilators without providing direct bronchodilation
	Bioavailability enhancer	Piperine significantly inhibited CYP enzymes and P‐glycoprotein, hence augmenting Cmax, AUC, and half‐life of various CYP‐substrate medications in humans. Extracts of black pepper and long pepper augmented the in vivo effects of diclofenac, alprazolam, and dexamethasone	Significant risk of pharmacokinetic interactions with oral corticosteroids, theophylline, some β‐blockers, leukotriene antagonists, and antihistamines (CYP/P‐glycoprotein substrates). Exhibits dose‐dependent effects, potentially enhancing both efficacy and toxicity

Abbreviations: AP‐1, activator protein 1; AUC, area under the curve; Cmax, maximum plasma concentration; CXCL, C‐X‐C motif chemokine ligand; ERK, extracellular signal‐regulated kinase; FEF, forced expiratory flow; FeNO, fractional exhaled nitric oxide; FEV, forced expiratory volume; HMGB1, high mobility group box 1; IFN‐γ, interferon‐gamma; IL, interleukin; JNK, c‐Jun N‐terminal kinase; KC, keratinocyte‐derived chemokine; LABA, long‐acting beta_2_‐agonist; MAPK, mitogen‐activated protein kinase; NF‐κB, nuclear factor kappa B; OVA, ovalbumin; PEF, peak expiratory flow; PEFR, peak expiratory flow rate; RAGE, receptor for advanced glycation end products; SABA, short‐acting beta_2_‐agonist; Th, T helper.

Some HMs showed the ability to not only mitigate inflammation but also to induce bronchial smooth muscle relaxation or prevent histamine‐induced bronchoconstriction. Specifically, *Tylophora indica*, *Adhatoda vasica* , and *Boswellia serrata* are documented to demonstrate bronchodilator effects that are either comparable to or supportive of β_2_‐agonists; thus, they hold promise for pharmacodynamic synergy with both SABA/LABA (Arora 2019; Gururani et al. 2023; Hewedy 2020). It is important to recognize that these effects primarily pertain to animal models and initial clinical trials.

From the viewpoint of the leukotriene pathways, it has been proposed that plants like *Curcuma longa* , *Astragalus membranaceus*, and *Glycyrrhiza glabra* inhibit 5‐lipoxygenase activity, leukotriene synthesis, and eosinophilic infiltration; thus, they could show a synergistic effect by suppressing the upper pathway through LTRA (D'Avino et al. 2023; Fouladi et al. 2019; Kim et al. 2024). This situation raises concerns regarding the potential for increased efficacy or the risk of side effects stemming from pharmacodynamic overlap. In contrast to biological agents (anti‐IL‐5, anti‐IL‐4/13, anti‐IgE), numerous herbal products inhibit cytokines via multiple immune pathways rather than a singular target, resulting in a broader yet less selective effect profile. This outcome emphasizes the promise of herbal products as economical adjuvants, especially in contexts with restricted access to biological therapies; yet, clinical evidence is evidently scarce.

The most significant discovery from a pharmacokinetic aspect is the strong inhibitory effects of formulations including *Piper nigrum* and piperine on CYP450 enzymes and P‐glycoprotein (Coelho et al. 2022). This feature elevates clinically significant HDI and DDI risks when used with anti‐asthma medications that are substrates of CYP or transporters, including oral corticosteroids, theophylline, certain antihistamines, and leukotriene antagonists. Consequently, the incorporation of piperine‐containing medicines in asthma therapy necessitates meticulous assessment about their possible advantages alongside the risks of dose‐dependent toxicity and interactions.

## 6 Clinical and Safety Considerations

The concurrent use of HMs with conventional anti‐asthma pharmacotherapy introduces a constellation of clinical risks that extend well beyond direct pharmacological interactions. Three interrelated domains—product standardization, adverse effect profiles, and healthcare‐professional engagement—collectively shape patient safety outcomes when botanical and conventional therapies intersect. Drawing on pharmacovigilance registries, analytical quality‐control studies, and recent clinical‐practice surveys, this section delineates the current landscape of safety considerations and identifies the practice gaps that most directly threaten asthma patients who combine these therapies.

### 6.1 Standardization and Dose Variability of Herbal Products

A fundamental challenge in evaluating HDIs lies in the compositional variability intrinsic to herbal products. Unlike synthetic pharmaceuticals manufactured under tightly controlled specifications, HMs are complex phytochemical matrices whose bioactive concentrations fluctuate according to plant genetics, geography, climate, harvest timing, post‐harvest processing, and extraction methodology (Wang et al. 2023; Zhang et al. 2012). Herbal product quality problems have been broadly categorized into external sources of variability, including contamination, adulteration, and misidentification, and internal sources of variability, such as phytochemical complexity and compositional non‐uniformity; together, these challenges substantially hinder the establishment of reproducible dosing regimens (Zhang et al. 2012). In response to these limitations, the WHO has published a series of technical guidance documents addressing marker‐substance selection, good chromatography practices, and quality standards for pharmaceutical quality‐control laboratories (WHO 2017, 2019a, 2024). Nevertheless, uptake of these standards varies markedly across regulatory jurisdictions: surveyed the regulatory landscape across the world and documented striking inconsistencies in pre‐market authorization, post‐market surveillance, and quality enforcement—an asymmetry that the recently adopted WHO Global Traditional Medicine Strategy 2025–2034 explicitly identifies as a priority for reform (Thakkar et al. 2020; WHO 2025).

The clinical implications of batch‐to‐batch variability are particularly acute for herbal products that modulate cytochrome P450 (CYP) enzymes, because the magnitude of enzyme induction or inhibition and therefore the risk of interaction with co‐administered asthma drugs depends directly on the concentration of bioactive constituents (Wanwimolruk and Prachayasittikul 2014; Zhou et al. 2003). St. John's wort ( *Hypericum perforatum* ) illustrates the problem vividly. Hyperforin, the principal CYP3A4 and PXR inducer in this botanical, ranges from 0.01% to 1.89% across commercial preparations in the United States, while hypericin content spans 47% to 165% of label claims (de los Reyes and Koda 2002). Other studies compared multiple batches from eight German manufacturers and observed pronounced interbatch variability that rendered products functionally non‐interchangeable (Wurglics et al. 2001). Because hyperforin content correlates directly with the degree of CYP3A4 induction, and because we have now demonstrated a clear dose‐dependent induction relationship in humans with CYP3A activity increasing up to 11‐fold at higher doses, this compositional drift translates into unpredictable effects on theophylline clearance, corticosteroid metabolism, and other CYP3A4‐dependent pathways critical to asthma management (Hohmann et al. 2024; Nicolussi et al. 2020).

*Ephedra sinica* , historically among the most widely used botanical treatments for respiratory disease, exhibits similarly concerning variability. The study analyzed nine commercial Ephedra‐containing supplements and reported ephedrine content ranging from 1.08 to 13.54 mg per dosage unit, a greater than 12‐fold variation, with one product exhibiting 137% lot‐to‐lot variability in alkaloid content (Gurley et al. 1998).

*Ginkgo biloba* products present analogous challenges. Although pharmacopoeial standards specify standardization to 22%–27% flavonol glycosides and 5.4%–6.6% terpene lactones, it has been estimated that roughly 70% of standardized Ginkgo extracts consist of poorly characterized constituents (van Beek and Montoro 2009). It is noteworthy that, an analysis of 14 commercially available finished Ginkgo products demonstrated that only three were authentic, while the others had been adulterated with exogenous rutin, quercetin, kaempferol, or isorhamnetin to fulfill pharmacopoeial quantitative requirements despite the absence of genuine Ginkgo‐derived flavonoids (Ma et al. 2016). This form of economically motivated adulteration may compromise the interpretability of clinical interaction studies, because Ginkgo extracts produced through different manufacturing processes can differ substantially in their phytochemical composition and, consequently, in their pharmacokinetic, pharmacodynamic, efficacy, and safety profiles (Hoerr et al. 2022; Kulić et al. 2022).

Among other plants relevant to asthma, *Nigella sativa* (black seed) also exhibits marked variability in thymoquinone content, with an analysis of 11 commercial products revealing a more than 260‐fold difference across samples (Khaikin et al. 2022). For *Boswellia serrata* , the limited oral bioavailability of 3‐O‐acetyl‐11‐keto‐β‐boswellic acid (AKBA), the principal 5‐lipoxygenase inhibitor, has raised concerns regarding its appropriateness as a quality marker, whereas subsequent analytical evidence has indicated that commercial claims of > 30% AKBA content are not phytochemically plausible, underscoring a persistent discrepancy between marketing statements and actual composition (Abdel‐Tawab et al. 2011; Mannino et al. 2016). Curcumin formulations present a distinct challenge for standardization, as the inherently poor oral bioavailability of native curcumin has led to the development of multiple proprietary delivery systems, including BCM‐95, Theracurmin, Meriva, and CurQfen, with substantially different bioavailability and pharmacokinetic profiles. Accordingly, products marketed generically as “curcumin supplements” may yield markedly different systemic exposures, and the evaluation of potential interaction risk should therefore be undertaken on a formulation‐specific basis rather than assumed from nominal curcumin content alone (Bučević Popović et al. 2024; Hegde et al. 2023; Sunagawa et al. 2015).

Beyond variability, herbal product quality is further threatened by outright contamination and adulteration. Evidence from an overview of 26 systematic reviews documented contamination with dust, pollens, microorganisms, fungi, toxic metals, pesticides, and undeclared synthetic drugs (Posadzki et al. 2013). An analytical survey from Taiwan cited in a subsequent review reported that 24% of Chinese herbal medicine samples contained at least one synthetic pharmaceutical, with corticosteroids among the prominent adulterants (Ernst 2002). This issue is particularly relevant to patients with asthma, who may unknowingly ingest synthetic glucocorticoids while assuming that they are using a natural therapeutic alternative (Ernst 2002).

### 6.2 Adverse Effects and Toxicity Reports

The toxicity profiles of medicinal plants used in asthma management span a spectrum from mild gastrointestinal disturbances to life‐threatening cardiovascular events, organ injury, and anaphylaxis (Choi and Shin 2024; Posadzki et al. 2013). Critically, many of these adverse effects are potentiated by concurrent use of conventional asthma medications through pharmacokinetic or pharmacodynamic interactions mediated by CYP450 enzymes, P‐glycoprotein, and shared receptor pathways (Zhou et al. 2003).

#### 6.2.1 Cardiovascular and Sympathomimetic Toxicity

*Ephedra sinica* is among the botanicals used in respiratory conditions with the most extensively documented cardiovascular safety concerns (Haller and Benowitz 2001). In a review of 140 adverse‐event reports submitted to the U.S. Food and Drug Administration between 1997 and 1999, 31% of cases were judged to be definitely or probably related to the use of ephedra‐containing supplements (Haller and Benowitz 2001). Among the events considered definitely, probably, or possibly related to ephedra exposure, 47% involved cardiovascular manifestations, including hypertension, palpitations or tachycardia, stroke, and myocardial infarction (Haller and Benowitz 2001). These concerns were reinforced by the subsequent accumulation of serious safety signals, culminating in the FDA's 2004 prohibition of ephedra‐containing dietary supplements. A more recent systematic review and meta‐analysis reaffirmed that even at lower therapeutic doses, ephedra‐containing preparations produce dose‐dependent increases in adverse cardiovascular outcomes, underscoring the need for vigilance when patients with asthma concurrently receive beta‐2 agonists (Cho et al. 2024).

#### 6.2.2 CYP‐Mediated Interactions and Theophylline Toxicity

St. John's wort represents the archetypal CYP‐mediated interaction risk for asthma patients (Henderson et al. 2002; Hohmann et al. 2024; Markowitz et al. 2003; Nebel 1999; Nicolussi et al. 2020). An early case described a patient on stable theophylline whose plasma concentrations fell below the therapeutic range after initiation of St. John's wort and recovered on discontinuation (Nebel 1999). Clinically significant interactions with theophylline, cyclosporine, warfarin, and oral contraceptives were subsequently catalogued, implicating induction of CYP3A4, CYP2C9, CYP1A2, and P‐glycoprotein (Henderson et al. 2002). In a controlled study of 12 healthy volunteers, 14 days of St. John's wort supplementation significantly increased CYP3A4 activity as probed by alprazolam pharmacokinetics (Markowitz et al. 2003). The clinical relevance of these interactions was later revisited, and hyperforin content was shown to correlate directly with the degree of CYP3A4 induction, implying that low‐hyperforin preparations may carry substantially lower interaction risk, a point that reinforces the product‐standardization issues discussed in Section 6.1 (Nicolussi et al. 2020). The most recent human dose–response data quantified up to an 11‐fold induction of CYP3A activity with escalating St. John's wort doses and observed effects comparable to those of rifampin, establishing the steepness of this relationship and its potential to compromise the therapeutic windows of narrow‐index drugs such as theophylline (Hohmann et al. 2024).

Among other CYP‐active herbs relevant to asthma, *Panax ginseng* has been studied for its effects on CYP‐mediated drug metabolism (Malati et al. 2012). In a controlled pharmacokinetic study of healthy volunteers who received 500 mg of *Panax ginseng* twice daily for 28 days, a significant decrease in midazolam plasma exposure was reported, consistent with induction rather than inhibition of CYP3A4 (Malati et al. 2012). This finding suggests that concurrent ginseng use may reduce systemic exposure to CYP3A4‐metabolized corticosteroids and theoretically compromise therapeutic efficacy (Malati et al. 2012). Interactions between seven top‐selling herbal products and prescribed medications were also documented, establishing the breadth of the herb–drug interaction problem across multiple botanical classes (Izzo and Ernst 2009).

#### 6.2.3 Antiplatelet Activity and Hemorrhagic Risk

*Ginkgo biloba* , occasionally used by asthma patients for its purported anti‐inflammatory properties, has been associated with spontaneous bleeding events (Bent et al. 2005; Kellermann and Kloft 2011; Mei et al. 2017). A systematic review identified 15 published case reports of bleeding temporally associated with Ginkgo use, including eight episodes of intracranial hemorrhage (Bent et al. 2005). A broader review of Ginkgo‐induced toxicity subsequently compiled both experimental and clinical evidence on this issue (Mei et al. 2017), Against these case‐series concerns, a meta‐analysis of 18 randomized controlled trials involving 1985 adults found that standardized Ginkgo extract (EGb 761) did not significantly increase bleeding risk (Kellermann and Kloft 2011). This discrepancy between case reports and controlled trial data may reflect confounding by non‐standardized products, concomitant anticoagulant use, or inter‐individual pharmacogenetic variability (Bent et al. 2005; Kellermann and Kloft 2011; Mei et al. 2017).

#### 6.2.4 Pseudoaldosteronism and Mineralocorticoid Excess

*Glycyrrhiza glabra* , a constituent of many traditional asthma formulations, carries a distinctive metabolic risk (Sabbadin et al. 2024; Yoshino et al. 2021). Its principal bioactive component, glycyrrhizin, is metabolized to glycyrrhetinic acid, which inhibits 11β‐hydroxysteroid dehydrogenase type 2 (11βHSD2) and produces pseudoaldosteronism characterized by hypokalemia, sodium retention, hypertension, and metabolic alkalosis (Yoshino et al. 2021). This mechanism is of particular concern in asthma patients receiving corticosteroids, as the combined mineralocorticoid effect may synergistically exacerbate potassium depletion, a risk that is further intensified by beta‐2 agonists promoting intracellular potassium shift; notably, high dosage, prolonged use, advanced age, hypoalbuminemia, and concomitant diuretic therapy have been identified as the principal risk factors for this complication (Yoshino et al. 2021). A recent case series further showed that even modest but sustained licorice intake, likewise through confectionery or herbal teas, can precipitate severe hypertension and hypokalemia in susceptible patients, highlighting the need for a routine dietary history in patients with uncontrolled asthma receiving corticosteroid therapy (Sabbadin et al. 2024).

#### 6.2.5 Hypersensitivity and Allergic Reactions

In atopic individuals, a population that substantially overlaps with patients with allergic asthma, *Echinacea* use warrants particular caution because immediate hypersensitivity reactions have been reported in association with its use (EMA 2008). The study reported that cases of anaphylaxis, acute asthma attacks, and urticaria/angioedema associated with *Echinacea* exposure, while skin‐prick positivity in *Echinacea*‐naïve atopic individuals further suggested that sensitization may arise through immunologic cross‐reactivity with other Asteraceae allergens (Mullins and Heddle 2002). This interpretation is supported by later safety reviews and by the European Medicines Agency (EMA), both of which acknowledge the risk of severe allergic reactions, including bronchospasm, asthma, and anaphylaxis, particularly in atopic patients or in those with hypersensitivity to plants of the Asteraceae family. Accordingly, in patients with atopic asthma, *Echinacea* should be approached with caution and may be considered relatively contraindicated, particularly in individuals with a history of pollen allergy, plant hypersensitivity, or prior immediate allergic reactions (Barnes et al. 2005; EMA 2008).

#### 6.2.6 Hepatotoxicity and Drug‐Induced Liver Injury

Herb‐induced liver injury has emerged as an increasingly recognized adverse effect (Byeon et al. 2019; Halegoua‐DeMarzio et al. 2023; Halegoua‐DeMarzio and Navarro 2025; Likhitsup et al. 2024; Lombardi et al. 2021). A synthesis of 31 studies encompassing 7511 cases of drug‐induced liver injury attributed 25% of cases to herbal products (Byeon et al. 2019).

Turmeric and curcumin, once viewed as unambiguously safe, have since become a focus of concern, with 10 cases reported from the United States Drug‐Induced Liver Injury Network (DILIN) showing latency periods of 1–4 months, marked hepatocellular injury, and evidence suggestive of an underlying immunogenetic predisposition (Halegoua‐DeMarzio et al. 2023). Additionally, studies show that the high bioavailability of curcumin formulations is identified as the common factor (Lombardi et al. 2021). Population‐level analyses based on nationally representative data have estimated that approximately 15.6 million United States adults used at least one of six botanicals with potential hepatotoxicity within a 30‐day period, with turmeric being the most commonly reported product (Likhitsup et al. 2024). *Nigella sativa* , generally considered safe at recommended doses, has nevertheless shown in vitro cytotoxicity and genotoxicity at higher thymoquinone concentrations, including GSH depletion and DNA damage in hepatocyte cultures (Khader et al. 2009; Tavakkoli et al. 2017). By contrast, *Boswellia serrata* appears to retain a favorable hepatic safety profile (Abdel‐Tawab et al. 2011).

### 6.3 Role of Healthcare Professionals in Preventing Herb‐Drug Interactions

Preventing herb–drug interactions in patients with asthma requires a coordinated, multidisciplinary approach across the continuum of care, as effective asthma management depends on the integration of evidence‐based strategies into clinical practice and the engagement of multiple stakeholders, while the interaction potential of herbal products must be explicitly considered during medication review and clinical decision making (Asher et al. 2017; Awortwe et al. 2019). In practice, however, three recurring barriers: low patient disclosure, inadequate provider knowledge, and fragmented clinical decision support limit the effectiveness of this safety net (Dores et al. 2023; Foley et al. 2019; Mehta et al. 2008; Shen et al. 2025).

#### 6.3.1 Patient Disclosure and the Hidden Prevalence of Herbal Use

The most fundamental barrier is that providers frequently remain unaware of their patients' herbal use (Foley et al. 2019; Kennedy et al. 2008; Mehta et al. 2008). A systematic review of 86 studies, pooling 14 quantifiable cohorts, reported a disclosure rate for biologically based complementary medicines of only 33% (Foley et al. 2019). An analysis of National Health Interview Survey data from 5196 herb and supplement users similarly found that only approximately one‐third disclosed their use to a physician, with particularly low disclosure rates among males, younger adults, and racial or ethnic minorities (Kennedy et al. 2008). Comparable figures were also reported among individuals with chronic conditions, with significant ethnic disparities in disclosure behavior (Mehta et al. 2008). The most commonly cited reasons for non‐disclosure include the perception that herbal products are inherently safe, anticipated disapproval from conventional providers, and, critically, provider failure to ask (Foley et al. 2019).

These general findings are amplified in the asthma population (Braunwalder et al. 2025; Rivera et al. 2004; Slader et al. 2006). A study reported that complementary and alternative medicine use ranged from 4% to 79% among adults with asthma and from 33% to 89% among children with asthma (Slader et al. 2006). The disclosure gap was particularly evident in one clinical study, where chart review identified no herbal use among asthma patients, while prospective interviews revealed that 42% were actively using herbal products, including several with the potential for adverse interactions with prescribed medications (Rivera et al. 2004). A recent systematic review of phytotherapy trials in bronchial asthma further indicated that patients frequently use multiple botanicals concurrently, thereby compounding the risk of unrecognized interactions and reinforcing the need for systematic practices that promote disclosure (Braunwalder et al. 2025).

#### 6.3.2 Knowledge Gaps Among Healthcare Professionals

Provider ability to recognize and manage herb–drug interactions is constrained by measurable knowledge deficits (Carr and Santanello 2019; Choudhury et al. 2023; Clement et al. 2005). In a survey of 127 United States community pharmacists, only 34% reported confidence in counseling patients about HMs, approximately half rarely initiated inquiries about herbal use, and only 19% consistently discussed potential herb–drug interactions when such use was disclosed (Carr and Santanello 2019). In a cross‐sectional survey of 192 physicians across six public hospitals in Trinidad, a marked gap between acceptance and knowledge was observed, with mean overall knowledge corresponding to approximately 15% of the maximum achievable score and only 15.1% of respondents able to identify at least one herb–drug interaction (Clement et al. 2005). These patterns recur across clinical settings and specialties, indicating a systemic educational deficit rather than an isolated shortfall (Choudhury et al. 2023; Clement et al. 2005). Choudhury et al. further framed the problem in terms of pharmacovigilance architecture, highlighting under‐reporting of adverse events, inadequate quality‐control infrastructure, and persistent patient–provider communication barriers as major obstacles to an effective safety system (Choudhury et al. 2023).

#### 6.3.3 Toward an Integrated Safety Framework

Several lines of evidence indicate that targeted educational interventions can measurably improve the clinical management of herb–drug interactions. Structured curricula on HMs and dietary supplements have been shown to produce significant and sustained gains in clinician knowledge and confidence (Beal et al. 2006). At the respiratory‐medicine level, herbal preparations have been described as the third most popular CAM modality among surveyed asthma patients, underscoring the practical relevance of equipping respiratory specialists with herbal‐medicine literacy; this remains directly relevant within contemporary asthma care planning frameworks, including the 2025 GINA strategy (Global Initiative for Asthma 2025; Huntley and Ernst 2000).

At the regulatory level, the WHO has progressively expanded its frameworks for traditional and complementary medicine. The WHO Global Report on Traditional and Complementary Medicine 2019 documented that 98 of 194 Member States had established national traditional medicine policies and that 124 countries had implemented regulations for HMs (WHO 2019b). The subsequently adopted Global Traditional Medicine Strategy 2025–2034 builds on these foundations and prioritizes quality, safety, and regulatory mechanisms for traditional, complementary, and integrative medicine (WHO 2025). A practical framework for preventing herb–drug interactions in asthma care should integrate routine screening for herbal use into every clinical encounter by employing standardized and non‐judgmental inquiry methods shown to improve disclosure rates; position pharmacists in a proactive gatekeeping role, particularly at the community pharmacy level where prescription and herbal purchases often converge; embed herb–drug interaction alerts within electronic health records linked to validated databases; incorporate evidence‐based modules on common herbs, their CYP450 interaction profiles, and the risks they pose to narrow‐therapeutic‐index drugs such as theophylline into continuing professional education; and, at the regulatory tier, require standardized labeling of bioactive constituent content, such as hyperforin in St. John's wort, ephedrine alkaloids in *Ephedra*, and glycyrrhizin in licorice, to enable meaningful clinical risk assessment (Foley et al. 2019; Tarn et al. 2015).

## 7 Conclusion and Future Directions

Asthma management continues to rely on well‐established pharmacotherapies; however, the growing use of medicinal plants introduces clinically relevant complexities that cannot be overlooked. As highlighted throughout this review, botanicals with anti‐inflammatory and bronchodilatory properties may interact with anti‐asthma drugs through multiple pharmacokinetic and pharmacodynamic pathways, leading to adverse outcomes such as theophylline toxicity, pseudoaldosteronism, cardiovascular events, bleeding risk, and hepatotoxicity. Despite substantial mechanistic evidence, the clinical literature remains limited and methodologically heterogeneous, preventing definitive conclusions on safety and efficacy when combined. A critical challenge lies in interindividual variability, particularly pharmacogenetic differences affecting drug metabolism. When such variability is combined with enzyme‐modulating phytochemicals, drug–herb–gene interactions may produce clinically unpredictable exposure levels. In this context, physiologically based pharmacokinetic modeling and pharmacogenetic stratification represent promising tools for translating mechanistic insights into clinically actionable risk predictions. Future research should prioritize high‐quality clinical trials, standardized methodological frameworks, and the integration of real‐world data to bridge the gap between experimental findings and clinical applicability. In parallel, regulatory harmonization, improved pharmacovigilance, and robust standardization of herbal products are essential to ensure safety and reproducibility. At the clinical level, routine and systematic screening for herbal use, supported by targeted decision‐support systems and multidisciplinary collaboration, will be critical to mitigate preventable risks. In conclusion, medicinal plants may complement conventional asthma therapies only when their potential for interaction is rigorously characterized and explicitly incorporated into clinical decision‐making. Advancing this field will depend on shifting from descriptive, fragmented evidence to predictive, integrative models that account for pharmacokinetic, pharmacogenetic, and real‐world clinical variability.

## Author Contributions

**Emine Kocyigit:** conceptualization, investigation, writing – original draft, visualization. **Aylin Bülbül:** writing – original draft, investigation, visualization. **Betul Kocaadam‐Bozkurt:** writing – original draft, investigation, visualization. **Osman Bozkurt:** writing – original draft, investigation, visualization. **Duygu Ağagündüz:** conceptualization, writing – review and editing, supervision. **Raffaele Capasso:** conceptualization, writing – review and editing, supervision.

## Funding

The authors have nothing to report.

## Consent

The authors have nothing to report.

## Conflicts of Interest

The authors declare no conflicts of interest.

## Data Availability

Data sharing not applicable to this article as no datasets were generated or analyzed during the current study.
